# Dynamics of jaw kinematics and fundamental frequency suggest vocal tract tuning in the indris’ song

**DOI:** 10.1371/journal.pcbi.1014781

**Published:** 2026-09-21

**Authors:** Filippo Carugati, Olivier Friard, Chiara De Gregorio, Daria Valente, Anna Zanoli, Valeria Ferrario, Lia Laffi, Silvia Leonetti, Valeria Torti, Elisa Protopapa, Longondraza Miaretsoa, Cristina Giacoma, Marco Gamba

**Affiliations:** 1 Department of Life Sciences and Systems Biology, Università di Torino, Turin, Italy; 2 Department of Psychology, University of Warwick, Coventry, United Kingdom; 3 Fondazione Zoom, Cumiana, Turin, Italy; 4 Groupe d’Étude et de la Recherche sur les Primates de Madagascar (GERP), Antananarivo, Madagascar; 5 Institut Supérieur de Technologie Régionale (ISTR), Manakara, Madagascar; Leiden University: Universiteit Leiden, NETHERLANDS, KINGDOM OF THE

## Abstract

The ability to modify the supralaryngeal vocal tract, particularly by adjusting mouth opening, can align formants and harmonics, thereby enhancing vocal intensity. This phenomenon, known as formant tuning, has been described in human singers as well as in various mammalian and avian species. However, little is known about its role in singing primates. In this study, we investigated whether vertical mouth opening in the indri (*Indri indri*), the only known singing lemur, covaries with the fundamental frequency (*f*_o_) of its vocalizations, a parameter directly related to the first formant in this species. We analysed 83 video recordings from 19 wild indris, using a markerless pose estimation model based on DeepLabCut. We extracted frame-by-frame measurements of mouth opening, calculated as the pixel distance between predicted landmarks on the upper and lower lips. These measurements were paired with *f*_o_ values extracted from corresponding audio tracks. A generalized linear mixed model revealed a significant positive relationship between vertical mouth opening and *f*_o_, indicating that vocalizations with higher frequency value are associated with greater jaw openings. These results provide the first direct, quantitative evidence that indris modulate their mouth configuration in coordination with *f*_o_, supporting the hypothesis that they can tune formants. This ability likely contributes to increased sound intensity and transmission range, enhancing long-distance communication in dense forest environments. Beyond improving our understanding of vocal production in singing primates, this study combines markerless pose estimation with frame-level acoustic analysis to quantify articulatory dynamics from field recordings, providing a multimodal approach for investigating the relationship between vocal tract configuration and acoustic output in animal communication.

## Author summary

In many animals, including humans, the shape of the vocal tract, and particularly how wide the mouth is opened, can influence the resonance properties of the voice, potentially amplifying sound and increasing its transmission range. This phenomenon, known as formant tuning, is well-documented in professional singers, but evidence in non-human primates remains scarce. Here, we investigated whether indris, a species of lemur known for their complex songs, adjust their mouth movements to influence the acoustic properties of their calls. Using video recordings of wild individuals and a pose estimation approach to track facial movements, we measured how widely indris open their mouths while singing and compared these movements with the fundamental frequency of their vocalizations. We found that indris open their mouths more when producing higher-pitched sounds. This coordinated adjustment suggests that they may actively modify their vocal tract to enhance sound transmission. Our study provides new evidence that singing primates can fine-tune their vocal production and introduces a method to study these mechanisms in wild animals using combined video and audio data.

## Introduction

Vertical mouth opening, defined as the vertical separation of the lips during vocal production, plays a crucial role in shaping the acoustic output in many species, including human and nonhuman primates [1,2]. Changes in vertical mouth opening alter the configuration of the vocal tract and impact its resonant frequencies, also known as formants [3], i.e., resonances in the vibrating air within the vocal tract that selectively enhance specific spectral components during phonation [4]. These relationships have long been recognized in acoustic theory, from early resonance models developed by Hermann von Helmholtz [5] to later articulatory-acoustic descriptions of speech production [6,7]. From a biomechanical perspective, the effect of mouth opening on the first vocal tract resonance can be explained by perturbation theory and sensitivity functions of the vocal tract resonator, which describe how local changes in vocal tract geometry influence resonance frequencies [8]. In particular, increasing jaw opening enlarges the cross-sectional area near the vocal tract exit, typically raising the frequency of the first resonance and thus the first formant (F1) in the radiated acoustic spectrum.

Experimental studies on speech articulation confirmed this relationship, showing that F1 increases with greater jaw opening [9,10]. Articulatory parameters such as tongue height and position also strongly influence formant structure, often even more than lip opening itself, and can affect both F1 and the second formant (F2), thereby shaping vowel identity and acoustic distinctiveness [11].

Studies on human singing have expanded our understanding of how wider mouth openings affect sound radiation efficiency, particularly at higher frequencies [12]. Story and colleagues [13] showed that lip configuration and mouth-opening shape affect sound intensity and spectral characteristics. These studies combined imaging techniques and acoustic analyses to show that F1 and F2 can change consistently with increased vertical mouth opening, and that although fundamental frequency (hereafter *f*_o_) is primarily governed by the tension and length of the vocal folds, articulatory settings like mouth opening can have indirect effects [14]. Classical singing techniques reveal an association between a higher *f*_o_, raised F1 frequencies, and increased vertical mouth opening [12]. Larger mouth openings typically raise F1 frequencies, which can help align vocal tract resonances with the harmonics of *f*_o_ [15]. This phenomenon, known as formant tuning or vocal tract tuning, refers to the deliberate alignment of a vocal tract resonance with a harmonic of the source signal in order to increase the radiated sound level. Formant tuning is a well-documented strategy in human singing, particularly in high-frequency vocal emissions, and has been described in detail in classical studies of singing acoustics [16–20]. When such alignment occurs, substantial increases in sound level can be achieved, as quantitatively demonstrated in a recent study [21].

A small number of studies have investigated whether formant tuning occurs in non-human mammals [22–24]. Some species display coordinated jaw and laryngeal adjustments that may accompany shifts in *f*_o_ [25], and affect F1 [3]. Especially those species in which projecting vocalisations at a distance can be critical for spacing and resource defence may alter the vertical mouth opening to modify the vocal tract’s shape, thereby affecting the size of the oral cavity and the position of the lips. This would influence F1 and favour the landing of the *f*_o_ (or its harmonics) on values similar to that of F1 [26]. When the *f*_o_ or one of its harmonics lands near F1, the vocal output becomes louder and clearer, as shown in macaques [27].

For instance, grey wolves (*Canis lupus hudsonicus*) can modify their mouth configuration during howling by lowering the jaw, thereby aligning the fundamental frequency (*f*_o_) with the first vocal tract resonance [22]. Similarly, grasshopper mice (*Onychomys* spp.) are able to tune *f*_o_ to the second resonance, thereby increasing the acoustic power of their long-distance calls [23]. Interestingly, the wapiti (*Cervus canadensis*) exhibits modulation of the source signal by a vocal tract resonance during the production of its biphonic, whistle-like sexual calls (“bugles”), suggesting dynamic adjustment of the supralaryngeal vocal tract configuration during vocal emission [28]. Beyond mammals, formant tuning has been described in several passerine species, including the Northern cardinal (*Cardinalis cardinalis*) and the white-throated sparrow (*Zonotrichia albicollis*) [29,30]. These studies demonstrate that birds can actively regulate the volume of the oropharyngeal-oesophagal cavity (OEC) through coordinated movements of the larynx and hyoid apparatus. Such adjustments modify the primary vocal tract resonance relative to the fundamental frequency (*f*_o_), thereby enhancing acoustic transmission efficiency [29,30].

Formant tuning is particularly interesting in the context of singing, but information on nonhuman primates producing songs is currently scarce. Singing is a rare behaviour that has evolved independently in four phylogenetically unrelated taxa, accounting for approximately 16% of all primate species [31–33]. Songs are distinctive series of vocalizations comprising different types of vocal units (or notes), often organized in phrases, which are modulated in frequency and uttered following a hierarchical order [33]. The unique acoustical and evolutionary properties of these utterances support the idea that primate songs represent a pivotal model for understanding the evolutionary roots of human language and musicality [34]. Although this potential has been widely expressed with studies revealing parallels between human language and primates songs in terms of coding efficiency (i.e., linguistic laws [35,36]), combinatorics [37,38], rhythm [39–46] and vocal development [46–48]), few investigations have been carried out concerning phonatory mechanism and vocal tract configuration during song production and their effect on *f*_o_ and formants [24,49].

The link between mouth opening and vocal output during song production was first documented by Haimoff [50], who observed that silvery gibbons (*Hylobates moloch*) tend to open their mouths wider when emitting higher-frequency sounds. Moreover, a pioneering study by Koda and colleagues [24] suggested that gibbons are capable of tuning their supralaryngeal vocal tract to amplify the propagation of song emission, in a manner analogous to professional singers. By recording a single white-handed gibbon (*Hylobates lar*) in both normal air and helium-enriched conditions, where the sound velocity increased, and resonance frequency tended to shift upwards, the authors observed that in helium, but not in air, the *f*_o_ was masked by the first harmonic. This result suggests that gibbons, as observed in trained human singers, can execute sophisticated movements of their vocal apparatus, including modulation of lip and jaw configuration, to optimize vocal tract resonances and facilitate sound propagation [24].

Although current findings support the hypothesis that singing primates can adjust their mouth configurations to enhance sound propagation during song emission, quantitative analyses linking vocal tract configuration to acoustic output remain scarce. Further investigation using quantitative approaches is therefore needed to better understand the potential role of formant tuning in these species. One of the primary challenges in this field is integrating acoustic parameters with anatomical and visual data. Given that most singing primates are endangered and inhabit restricted and remote areas, it is challenging to obtain both anatomical samples, such as vocal tract casts, and high-quality video recordings of individuals engaged in natural song production [51,52]. Moreover, extracting information from video footage is highly time-consuming and has traditionally relied on discrete, categorical labels rather than continuous quantitative measurements [53]. Recent advances in artificial intelligence have opened new avenues for studying animal behaviour, particularly in quantifying movement [54,55]. Markerless pose estimation algorithms, such as DeepLabCut, enable researchers to identify specific body landmarks in raw videos, allowing precise description and tracking of posture and movement [56,57]. This approach could enable researchers to estimate distances among predicted key-points, such as the degree of mouth opening, as continuous variables. These variables can then be paired with spectral parameters extracted from synchronized audio recordings, providing a quantitative, multimodal framework for studying articulatory mechanisms and their relationship with vocal output.

This work aims to investigate the dynamics of mouth opening in wild indris (*Indri indri*) to determine whether this species has the potential for formant tuning. Indris are lemurs endemic to the northeastern rainforests of Madagascar, where they live in small family groups that occupy and defend portions of the forest through their characteristic songs [58–61]. Given that indris deliver a song in which frequency modulation occurs regularly and repeatedly [41,62,63], the species represents an ideal model for illustrating the co-occurrence of vertical mouth opening and the modulation of acoustic outputs, namely *f*_o_, in the context of long-distance communication. However, because indris do not survive in captivity and are confined to isolated forest patches, obtaining anatomical data for this species remains exceptionally challenging.

In this context, Carugati and colleagues [64] introduced a methodological pipeline for mapping facial configurations based on the distances between predicted facial landmarks across different primate species. This includes a model specifically developed for detecting facial landmarks in indris. Leveraging this approach, we can automate the extraction of a quantitative, frame-by-frame estimate of mouth opening from video footage of singing indris and pair it with *f*_o_ values obtained from corresponding audio tracks. We hypothesized that we should observe variation across mouth opening configurations and acoustic output if the indris exploit vertical mouth opening for acoustic advantage, enhancing acoustic propagation [65,66]. This prediction is grounded in prior findings by Gamba and colleagues [65], who reported variation in average *f*_o_ and F1 across different degrees of mouth opening. However, the authors visually ranked the degree of mouth opening into discrete categories and found that higher *f*_o_ was associated with mouth opening exceeding 30 degrees. On the contrary, if the articulatory effort is independent of the acoustic output, we would expect that *f*_o_ and vertical mouth opening would not correlate, showing that any configuration may occur at any frequency. While previous work on vocal tract tuning in primates has relied on a single female gibbon [24], our current investigation involves both male and female indris. In indris, sex-related variation in fundamental frequency may emerge depending on the type of vocal unit (i.e., note type) produced during the song [67,68]. To ensure that our findings can be robustly interpreted as species-level phenomena that are not sex-specific, we have structured our analyses to explicitly account for potential sex-related variation.

In summary, this study aims to provide the first quantitative evidence of formant tuning in the only singing lemur, *Indri indri*. Furthermore, it introduces a novel methodological approach to quantify articulatory behaviour in a rare, wild primate using video footage. By integrating visual and acoustic data, this framework paves the way for broader applications in the study of multimodal communication and vocal production mechanisms across taxa.

## Materials and methods

### Ethics

The non-invasive methods used for the data collection of this study adhere to the American Society of Primatologists (ASP) ‘Principles for the Ethical Treatment of Non-Human Primates’. Field data collection protocols were reviewed and approved by Madagascar’s Ministère de l’Environnement, de l’Ecologie et des Forets, under Permits: 2008: N°258/08/MEFT/SG/ DGEF/DSAP/SSE; 2009: N°243/09/MEF/SG/DGF/DCB.SAP/SLRSE; 2010: N°118/10/MEF/SG/DGF/DCB.SAP/SCBSE and N°293/10/MEF/SG/DGF/DCB.SAP/SCB; 2011: N°274/11/MEF/SG/DGF/DCB.SAP/SCB; 2012: N°245/12/MEF/ SG/DGF/DCB.SAP/SCB; 2013: permit not required as data collection was performed by Malagasy citizens only; 2014: N°066/14/MEF/SG/DGF/DCB.SAP/SCB; 2015: N°180/15/MEEMF/SG/DGF/DAPT/SCBT; 2016: N°98/16/MEEMF/SG/DGF/DAPT/SCB.Re and N°217/16/MEEMF/SG/DGF/DSAP/SCB.Re; 2017: 73/17/MEEF/SG/DGF/DSAP/SCB.RE; 2018: 91/18/MEEF/SG/DGF/DSAP/SCB.Re; 2019: 118/19/MEDD/SG/DGEF/DSAP/DGRNE and 284/19/MEDD/SG/DGEF/DSAP/DGRNE; 2019/2020: 338/19/MEDD/SG/DGEF/DSAP/DGRNE; 2022: 186/22/MEDD/SG/DGGE/DAPRNE/SCBE.Re 2023: 084/23/MEDD/SG/DGGE/DAPRNE/SCBE.Re and 399/23/MEDD/SG/DGGE/DAPRNE/SCBE.Re 2024: 161/24/MEDD/SG/DGGE/DAPRNE/SCBE.Re. Field data collection protocols were also approved by GERP (Groupe d’Etude et de Recherche sur les Primates de Madagascar), the association overseeing research in the Maromizaha New Protected Area.

### Data collection and preparation

We collected 83 video recordings of indri singing by sampling eleven groups of wild indris (for a total of 19 individuals, 11 females and 8 males) across multiple expeditions conducted between 2008 and 2024 in Eastern Madagascar, specifically in the Maromizaha forest (18° 56’ S, 48° 27’ E) and the Mitsinjo reserve (18° 56’ S, 48 ° 25’ E). We followed one group daily during each annual field campaign (approximately 5.43 ± 2.38 months per year), approximately from 6:00 AM to 1:00 PM. We recorded the animals opportunistically, with particular effort to frame the subject’s face whenever visible while singing. We recorded the indris at a distance ranging between 10 and 25 metres. Consequently, the spatial resolution of mouth movements varied across recordings depending on the distance from the subject and the framing of the face, and this factor may introduce some degree of quantization noise, which represents an inherent limitation of video-based measurements under naturalistic field conditions. Moreover, song emission is a behaviour whose occurrence varies through day and seasons [69,70], often occurring at the higher layers of the canopy, with an estimated cue count rate (i.e., the probability of song emission per hour) of 0.358 [71]. High-quality video footage was scarce due to the dense forest canopy, the arboreal behaviour of the indris, and challenging climatic conditions, including light, humidity, and rain.

We visually inspected all video footage using the open-source BORIS [72] to identify clips in which the singer’s face was fully visible for at least a second. We manually labeled the beginning and end of each vocal emission of the focal animal, using annotations in BORIS that we later extracted as TextGrid files [72,73]. Since the selected videos were recorded using different cameras over the years, we equalized the format, framerate, and file size with the FFmpeg framework [74], converting the files to the MP4 format with a framerate of 25 fps and a resolution of 960x540 pixels.

### DLC model development

**Fig 1:**
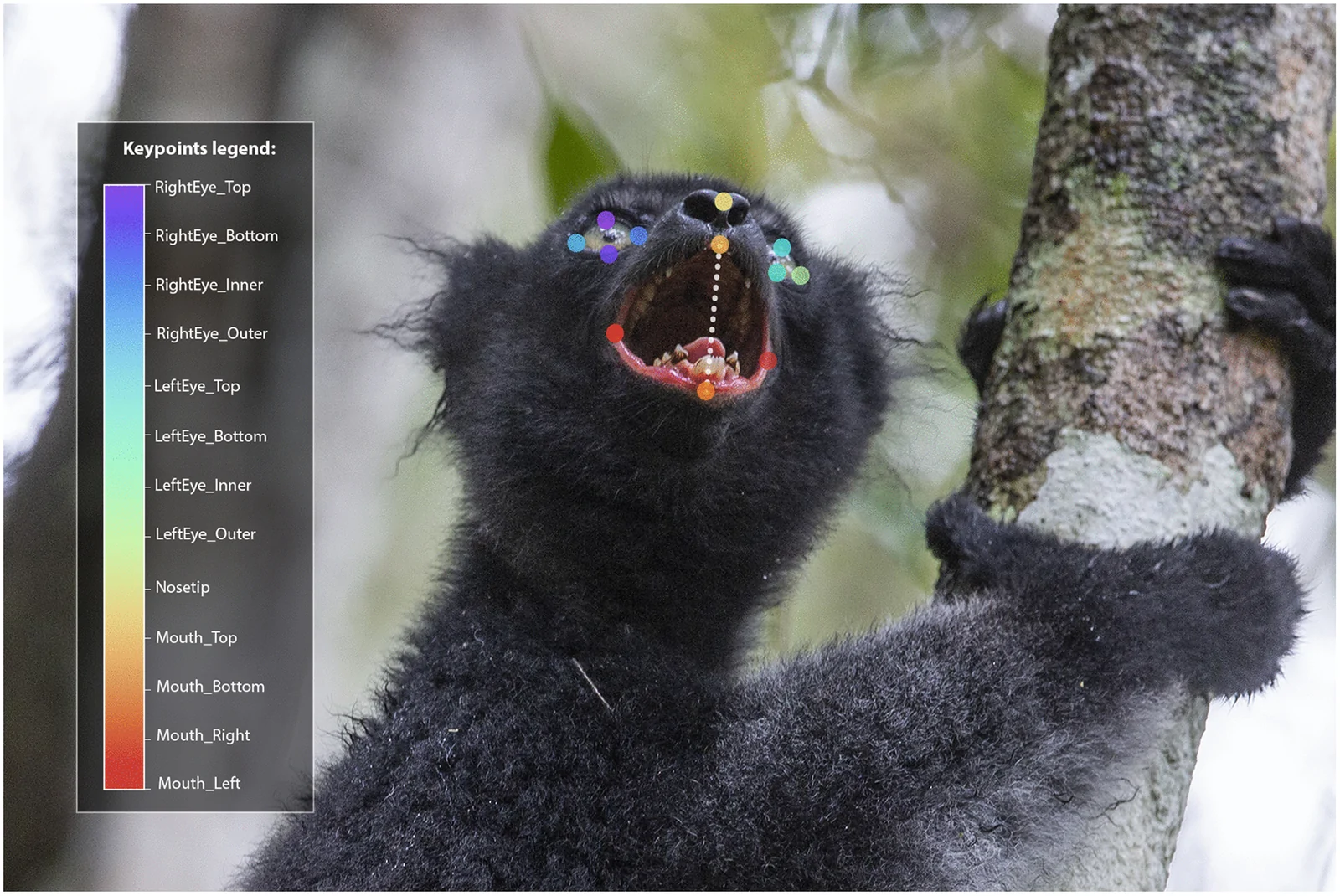
Set of 13 facial landmarks defined by Carugati et al. [64] for quantifying facial configuration in indris. The dotted line indicates the vertical mouth opening, calculated as the Euclidean distance between the Mouth_Top and Mouth_Bottom landmarks.

To quantify mouth shape from the video frames, we employed the DeepLabCut Python toolbox (version 2.3.8), hereafter referred to as DLC [56,57]. DLC is an open-source software that enables researchers to develop markerless pose estimation models that recognize user-defined sets of anatomical landmarks positioned on the target animal. For this study, we adapted the pre-existing DLC model, which we initially trained to identify 13 points on an indri face [64]. The first training used 2,355 labeled frames containing indri faces. It could recognize points distributed around the eyes, on the nose tip, at the centre of the upper lip, at the centre of the lower lip, and the mouth corners (Fig 1). To increase the accuracy of mouth points [75], we manually labeled the 13 landmark positions on 630 new frames extracted from 63 new videos framing indris during song emission. We then retrained the model, including the newly labeled images, using 95% of the frames for training and 5% for testing. For model training, we selected a ResNet-50-based convolutional neural network [76,77] with 1,300,000 iterations and a 0.9 p-cutoff (threshold for the likelihood of correct pose estimation). After model training, we evaluated its performance using the designed function on the DLC interface, yielding an RMSE (root mean square error) of 4.55 pixels in the test set (4.36 pixels if the p-cutoff is applied). We then used the DLC function *analyze_videos* to extract a CSV file for each frame, listing the coordinates of the predicted key points (along with their probability of correct estimation). A prediction with a probability below the p-cutoff for a given landmark resulted in an NA at its corresponding coordinates. Using a Python script (S1 File), we transformed the coordinates into distance matrices by computing the Euclidean distance between each pair of landmarks. All the normalized matrices were imported into the R software [78] and tabulated into a dataset of 77 nonredundant distances. From this dataset, we retained only the distance between the Mouth_Top and Mouth_Bottom landmarks, hereafter referred to as vertical mouth opening (represented by a dotted line in Fig 1), and the inter-ocular distance (RightEye_Inner-LeftEye_Inner). To contextualize the scale of the DLC model error, we compared it with the size of these anatomical features in the analysed frames. In the final dataset, the mean interorbital distance was 45.5 ± 17.0 pixels, and the mean vertical mouth opening was 50.6 ± 23.2 pixels. Thus, the model error corresponds to approximately 9–10% of the scale of the tracked features.

### Acoustic and video data processing

**Fig 2:**
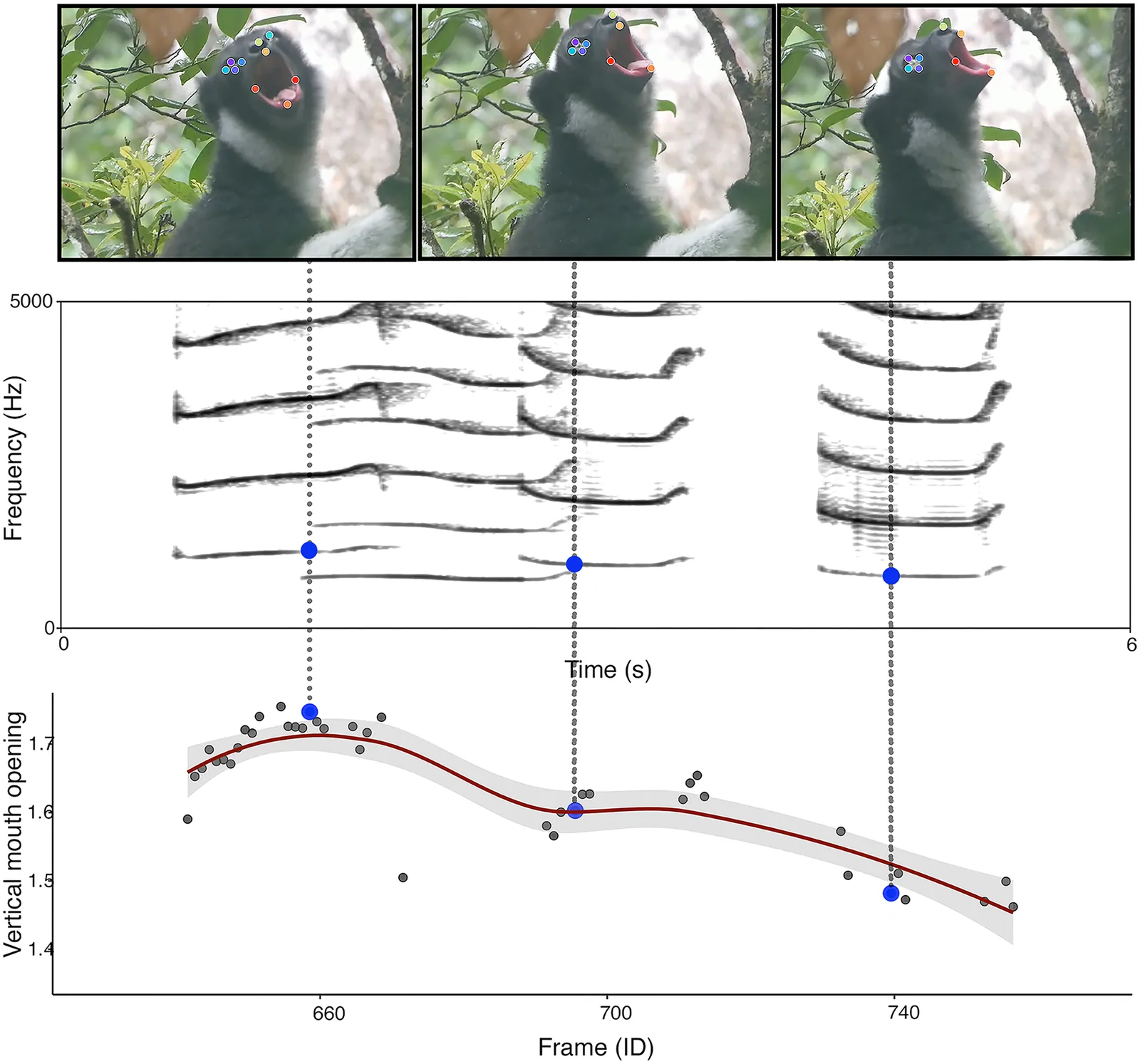
Visual representation of the alignment of visual and acoustic parameters during indris’ song production. The top panels show three frames with the predicted landmarks extracted from a single video clip. The middle panel displays a corresponding spectrogram, with blue dots indicating the estimated *f*_o_ values at the time points of the selected frames. The bottom panel shows a scatter plot of vertical mouth-opening measurements across the voiced frames in the same sequence. Blue points highlight the three frames shown above. The red line represents the estimated trend in mouth opening over time, while the grey shaded area indicates the 95% confidence interval.

We extracted audio files using FFmpeg [74] from the 83 original video recordings (PCM WAV format), preserving the original audio sampling rate (48000 Hz). We used a Praat script [79] (S1 File) to silence non-target audio sequences (e.g., silence between units and utterances by non-focal individuals) based on the TextGrid annotation. We then extracted *f*_o_ measurements using the cross-correlation pitch detection algorithm in Praat (“To Pitch (cc)”) with the following settings: time step = 0.001 s, pitch floor = 500 Hz, pitch ceiling = 1350 Hz, silence threshold = 0.03, voicing threshold = 4.5 × 10^-5^, octave cost = 1 × 10^-13^, octave-jump cost = 3.5 × 10 ^-13^, voiced/unvoiced cost = 1.4 × 10^-5^. We then joined the distances between key points and the *f*_o_ values using the *left_join* function (package *dplyr* [80],). From an initial dataset of 140,981 observations, we retained 11,467 matrices by excluding those in which either *f*_o_ or the vertical mouth opening could not be computed. The accuracy of key placements was then reassessed by visualizing their position (using the DLC *create_labeled_videos* function) and manually eliminating 2106 additional frames (corresponding to 18% of the dataset). In those frames, incorrect positioning could be caused, for instance, by the presence of branches or leaves overlapping the indri’s face. Fig 2 provides a visual overview of the multimodal alignment between visual and acoustic parameters, specifically vertical mouth opening estimates and *f*_o_ measurements.

Given that, as mentioned above, we recorded the animals at varying distances, we normalized the vertical mouth opening using the interocular distance (RightEye_Inner-LeftEye_Inner, following [64,75]). We note that interocular distance is a two-dimensional projection of a three-dimensional anatomical structure and may vary with head orientation relative to the camera. In particular, as the head rotates away from a frontal view, the projected interocular distance is expected to decrease non-linearly, potentially affecting this normalization procedure. Given the pronounced snout morphology of indris, even moderate deviations from frontal orientation typically reduce the visibility of key landmarks. As a result, the retained dataset is enriched with frames that approximate near-frontal head orientations, thereby limiting but not eliminating the magnitude of projection-related distortions.

As the interocular distance was not available for all frames in which we had both vertical mouth opening and *f*_o_, we computed the rolling mean of the interocular distance over a 30-frame window (15 frames before and after each target frame, i.e., 0.6 s before and after a target frame) using the *rollapply()* function from the package *zoo* [81]. This window size was chosen as a compromise between increasing the probability of retrieving reliable neighbouring estimates of the interocular distance and maintaining the smoothing procedure within the temporal scale of a single vocal unit. Individual notes in indri songs typically last between 1 and 4 s [36,82]; therefore, a ± 0.6 s window remains well within the duration of a single note. We normalized the vertical mouth opening with the rolling mean of the interocular distance, but only for cases where the coefficient of variation (hereafter, CV) for the interocular distance did not exceed 10% within the same video. When the CV exceeded 10%, indicating greater variability in apparent head size likely caused by changes in head orientation or camera perspective, we used the average rolling mean as the normalization factor. We excluded from further analyses those frames for which interocular distance was unavailable within a 30-frame window. The resulting dataset included 3598 frames with normalized vertical mouth opening and *f*_o_ measurements, retained from 23 videos of 12 individuals (seven females and five males - see S1 Table). We inspected the *f*_o_ contours of all units and manually corrected 74 of the 3598 instances (corresponding to 2.03% of the sample) in which the Praat function estimated the *f*_o_ values inaccurately. These inaccuracies were typically due to extraction errors in noisier segments of the units, often occurring at the ends of notes.

### Vocal tract models and estimation of formant variation with respect to the fundamental frequency

The scarcity of specimens and rigorous anatomical investigations prevented a detailed, anatomically faithful reconstruction of the indri’s vocal tract. We generated a concatenated tube computational model of the indri vocal tract by measuring and manipulating two indri skull replicas. The first replica was a high-quality polyurethane resin cast of a skull found in the Analamazaotra Special Reserve in 2003. The second was a commercially available resin cast (Skulls Unlimited, Bone Clones, Replica Indri Skull SKU BC-282). We averaged the two values to proceed with the modelling. We used variable mandible positioning to estimate vocal tract cross-sectional areas at mouth openings of 0.2 cm, 1.0 cm, 2.0 cm, 3.0 cm, 4.0 cm and 5.0 cm. This was measured as the distance from the top of the right central incisors of the maxilla and the mandible. We used a non-toxic compound composed of water, salt, wheat flour, and mineral oil to fill the cavities. This created a cast simulating the vocal tract areas of an open mouth from the back of the palate. We measured the cross-sectional areas at 0.5 cm intervals from the glottis to the tract’s end [83]. We also simulated the back cavities by modelling a uniform tube. This method was consistent with Milne-Edwards’s drawings in Grandidier (1875) [84]. Using measurements of indri skulls coming from different locations (N = 13, mean = 103.22 mm, sd = 2.68 mm) from the Dead Animal Collection of the Tsimbazaza Zoo in Antananarivo, we estimated a variation in greater skull length of approximately 7%. Following this, we modelled the vocal tract by simulating a 10% increase in the length and cross-sectional area of the tubes. We then collected the first three formants (F1, F2, F3) from the acoustic responses of each tube using the VTAR modelling software [85,86]. Formants were averaged by normalised mouth opening (simulated mouth opening/inter-orbital distance). This allowed us to compare, using a Spearman correlation, the estimated formants with fundamental frequencies measured from utterances with varying mouth opening. For interpretation, we calculated multiples H2 (*f*_o_*2), H3 (*f*_o_*3), H4 (*f*_o_*4), and H5 (*f*_o_*5) to determine whether formant tuning involved only the fundamental frequency or also higher harmonics. We plotted these against estimated formant variation.

### Statistical analysis

To investigate the relationship between normalized vertical mouth opening and *f*_o_, we fitted a generalized linear mixed model (GLMM) using the *glmmTMB* package [87] (S1 File) We assessed the distribution of the response variable using the *fitdistrplus* package [88], which indicated that a *beta* distribution was the most suitable. Consequently, *f*_o_ values were scaled to fall within the 0–1 interval to meet the distributional assumptions of the model. In the GLMM, *f*_o_ was the response variable and normalized vertical mouth opening and sex were the fixed factors. We inserted sex as a predictor following previous evidence of sexual differences in particular song units [68]. We used individual ID and the video filename as random factors. We created a full model with fixed and random effects, whereas the null model included only random effects. We performed model comparison using a likelihood ratio test via the *anova* [89] function to assess the significance of the fixed effects. We used the DHARMa package [90] to simulate residuals from the full model, allowing us to visually inspect their distribution. Then, we assessed whether actual and simulated residuals had equal dispersion.

In order to understand whether there was a correlation between the values of the fundamental frequency and the formants measured from the acoustic responses generated by the vocal duct models, we extracted the values relating to the same mouth openings after an interpolation of the model results. We performed a Spearman correlation test because both data sets showed significant deviations from normality.

## Results

**Fig 3:**
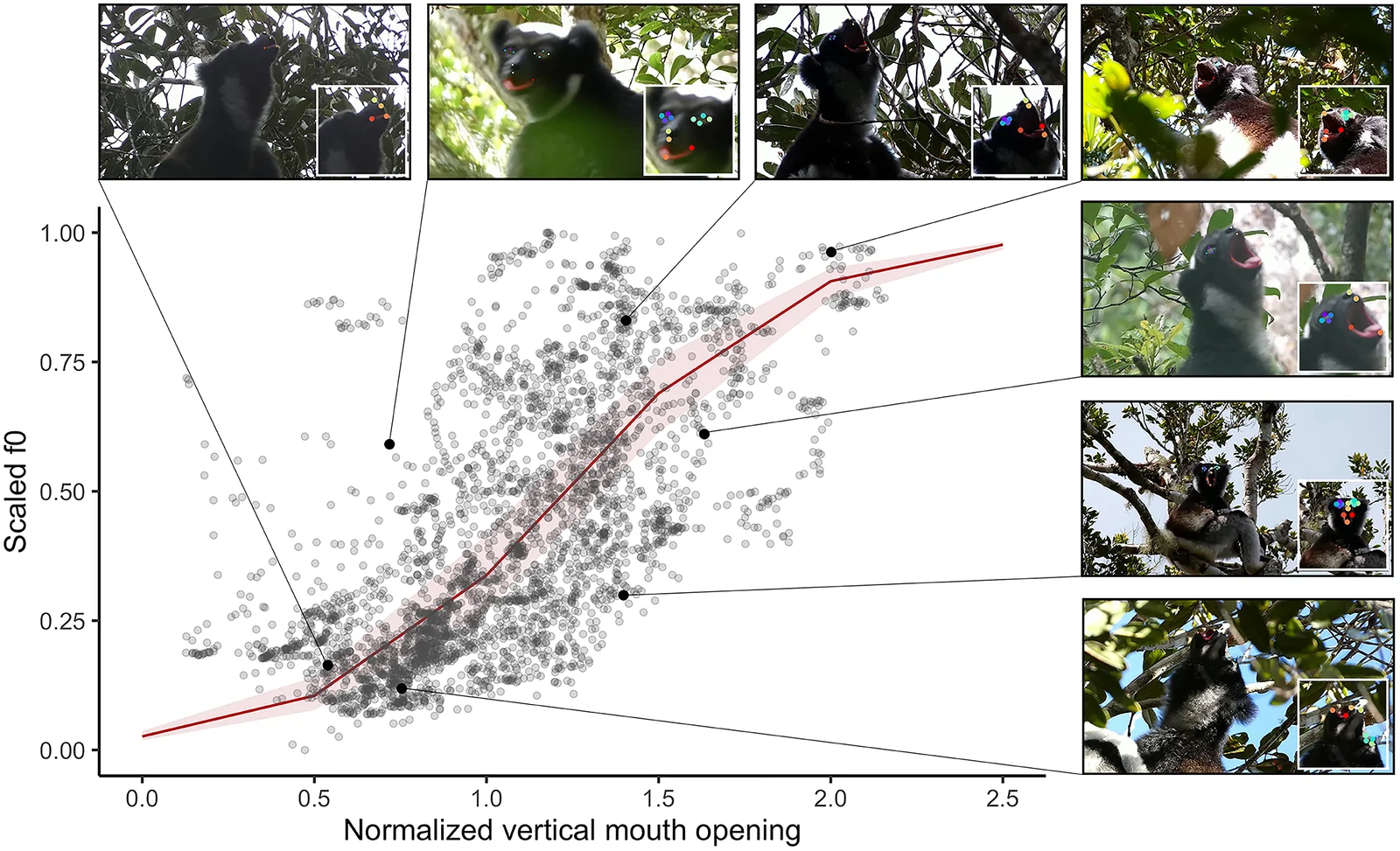
Effect of the normalized vertical mouth opening on the *f*_o_, originally spanning from 573 Hz to 1238 Hz. Red shaded areas indicate 95% confidence intervals. Dots represent the observed data, with larger and darker dots corresponding to example labeled frames visualized in the panels above and on the right of the plot. For each panel, a zoomed inset helps better visualize the position of the landmarks.

Measurements of normalized vertical mouth opening ranged between 0.13 (corresponding to 628 Hz) and 2.16 (1154 Hz). Overall, *f*_o_ estimates ranged from 573 to 1238 Hz. The full model significantly differed from the null model (χ² = 2520.8, df = 2, p < 0.001), indicating that sex and vertical mouth opening made a significant contribution to explaining variation in *f*_o_. In particular, we found that vertical mouth opening had a significant positive effect on *f*_o_ (p < 0.001; see Table 1), supporting that an increase in mouth opening is associated with higher *f*_o_ values. Conversely, we found no significant differences between males and females (p = 0.23), suggesting that our data did not show a sexually dimorphic variation of *f*_o_. Model results are summarized in Table 1. To visualize the relationship between normalized vertical mouth opening and scaled *f*_o_, we plotted the marginal effect predicted by the GLMM using the function *plot_model()* from the R package *sjPlot* [91]. Observed data points were overlaid on the predicted relationship (Fig 3). Simulated residual plots did not deviate from the model assumptions and showed no significant dispersion (dispersion = 0.999, p = 0.872).

**Table 1:** Model summary showing the influence of vertical mouth opening and sex to the *f*_o_.

Model: *f*_o_ ~ Vertical Mouth Opening + Sex + (1|individual ID) + (1|video filename)				
Predictors	Estimate	SE	z-value	p-value
(Intercept)	-3.717	0.213	-17.43	<0.001
Vertical Mouth Opening	**2.939**	**0.046**	**63.12**	**<0.001**
Sex (Male) ^a^	0.418	0.348	1.200	0.230

a. This predictor was dummy-coded, with ‘Sex (Female)’ being the reference category.

**Fig 4:**
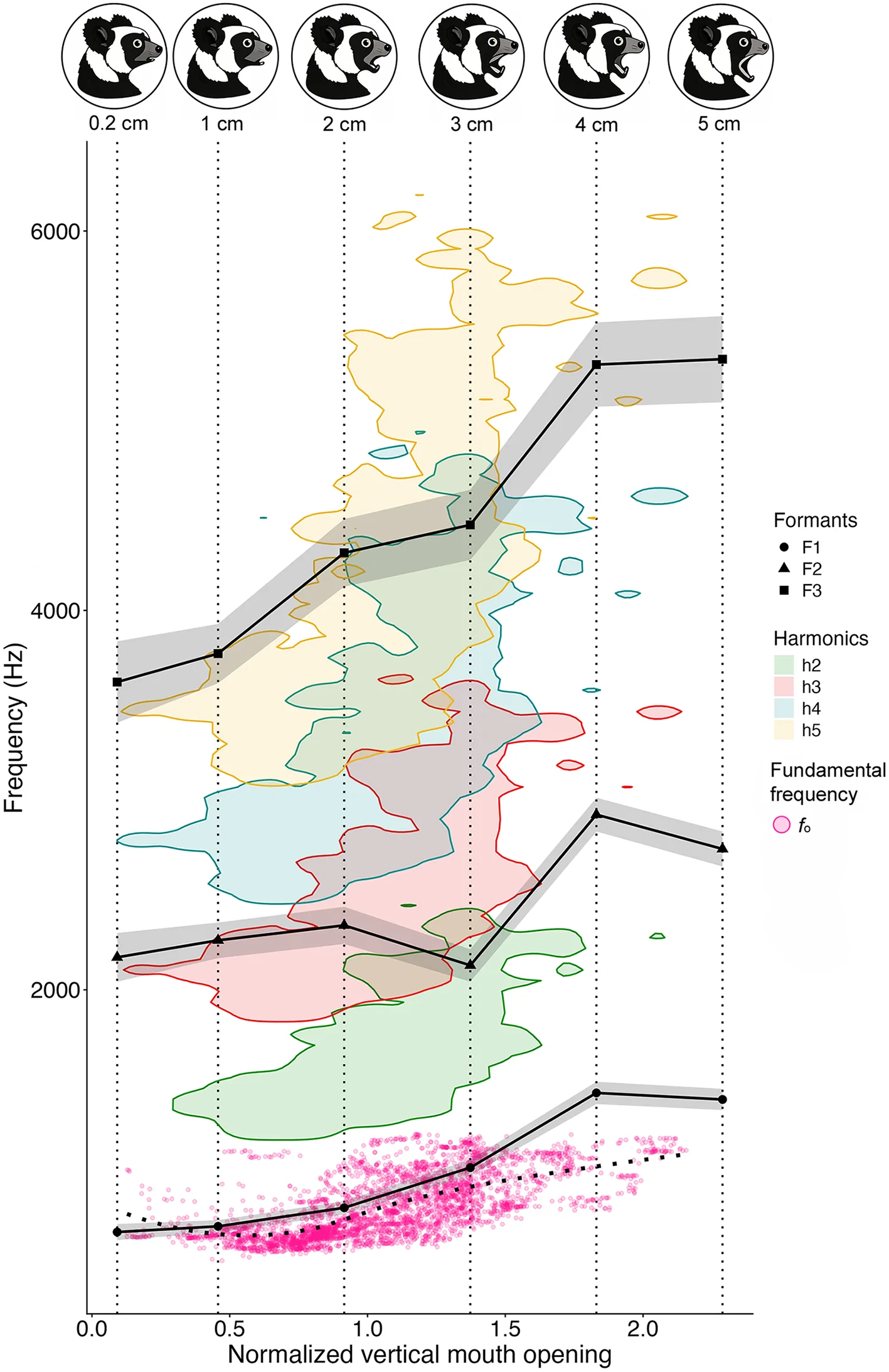
Relationship between normalized vertical mouth opening and spectral features. Pink points represent the fundamental frequency **(*f***_**o**_). Black lines with symbols denote the first three formants (F1, F2, F3), with shaded areas indicating variability. Coloured contours show the distribution of higher harmonics (H2–H5). Illustrations at the top depict increasing mouth opening (0.2–5 cm). Increasing mouth opening is associated with a systematic rise in formant frequencies and shifts in harmonic distribution.

We grouped the formants we extracted from the acoustic responses of the vocal tract models according to the different vertical mouth openings observed during the video analysis. We plotted the *f*_o_, the calculated harmonics (H2-H5), and the formants F1-F3 in Fig 4. We found significant Spearman correlations between *f*_o_ and the estimated values of F1-F3 for the corresponding vertical mouth opening positions (N = 3599; F1: rho = 0.698, p < 0.001; F2: rho = 0.039, p = 0.021; F3: rho = 0.698, p < 0.001).

## Discussion

To test the hypothesis that indris modify their vocal apparatus to maximise the projection of their song units, we quantified vertical mouth opening from video recordings and paired these measurements with instantaneous fundamental frequency (*f*_o_) values extracted from the corresponding audio tracks. We found a significant positive relationship between mouth opening and *f*_o_, indicating that indris adjust their mouth configuration in relation to the frequency of their vocal output.

### Technical considerations on vertical mouth opening estimation

In this study, we used markerless pose estimation to obtain quantitative measures of vertical mouth opening during song production. This approach extracts articulatory dynamics during vocal emission directly from raw video footage of wild, free-ranging primates. Through the application of deep learning algorithms, this workflow enables the automation of the information extraction process, greatly reducing the time needed for manual scoring. By combining these visual measurements with synchronised *f*_o_ estimates, we embraced a multimodal framework. This is especially useful for studying the evolution of primate communication and vocal production [92].

Despite these advantages, several methodological issues should be considered. We built on a pre-trained DeepLabCut model for indri facial landmark detection and manually labelled a limited number of additional frames to improve performance on singing sequences. Overall, model accuracy was similar to that in other DeepLabCut studies on wild primates [64,93]. The test error of 4.55 pixels was much smaller than both the mean interorbital distance and the mouth opening in our analysed frames (45.5 and 50.6 pixels, respectively), and also smaller than the observed biological variation in mouth opening. This suggests that tracking error is unlikely to have driven the main pattern reported here.

Nonetheless, visual inspection revealed occasional misplacements of landmarks. These were often caused by branches, leaves, or incorrect predictions on the upper or lower jaw. Such cases required manual verification and exclusion, which reduced the degree of workflow automation. This difficulty likely reflects the challenging recording conditions. Indris usually sing high in the canopy, orienting their heads in different directions to better broadcast their signals [94], often partly obscured by vegetation and subject to variable lighting and viewing angles. Future improvements should concentrate on expanding the training dataset to include more labelled images and recording conditions.

A further limitation arises from the need to estimate the movements of a three-dimensional structure from two-dimensional video. Both mouth opening and interorbital distance were derived from 2D facial landmark projections, so they may be affected by the head’s orientation relative to the camera. As the head rotates away from a frontal view, estimated distances between landmarks are expected to decrease non-linearly. This likely affects both raw measurements and normalisation. In practice, these effects were likely reduced, though not eliminated, because we excluded frames with substantial head rotation or occlusion. The retained dataset was thus biased toward near-frontal views. Normalisation by interorbital distance reduced variation due to camera distance and scale, though it did not fully correct for projection artefacts. Overall, perspective effects probably introduced more residual noise than they altered the relative variation in mouth opening across frames.

Further work could address this issue by combining markerless pose estimation with three-dimensional reconstruction, head-pose estimation, or simplified cranial models. These developments would yield a more accurate and generalizable framework for extracting articulatory information from field recordings and would strengthen multimodal analyses of primate communication.

### Do indris and other animals tune their vocal tract?

Our model showed that vertical mouth opening has a significant positive effect on *f*_o_, supporting the hypothesis that these parameters covary during indri song. This finding is consistent with earlier investigations by Gamba and colleagues [65], who reported that notes produced with wider mouth openings had higher average *f*_o_ and higher formant values, especially the first formant (F1, which is a resonance frequency of the vocal tract). Together, these results support the view that indris modify the supralaryngeal vocal tract (the part of the throat and mouth above the vocal cords) during song production and that increased jaw opening contributes to changes in vocal output.

Because tuning F1 (the first vocal tract resonance) to *f*_o_ or its harmonics can enhance vocal intensity, this pattern is compatible with the hypothesis of formant tuning (adjusting the vocal tract so that its resonance frequencies match the pitch or its harmonics). In ecological terms, such a mechanism may be advantageous in a species that relies on long-distance song to advertise territorial occupancy in dense forest habitat.

A limitation of our study is that we cannot directly demonstrate active or deliberate formant tuning by indris. However, the strong correlation between *f*_o_ and the modelled values of F1 and F3 provides robust support for a coupling between source and filter parameters mediated by vocal tract configuration. Importantly, the modelling results demonstrate that the observed range of mouth opening is sufficient to produce systematic shifts in formant frequencies that can align with the fundamental frequency or its harmonics. It should be noted, however, that according to perturbation theory a given resonance can be affected by changes in vocal-tract geometry at multiple positions along the tract. Thus, while our results are consistent with mouth opening as a driver of F1 variation, we cannot rule out additional or alternative contributions from other supralaryngeal adjustments, such as pharyngeal configuration or lip posture. The comparison with harmonic structure (H2–H5) further indicates that such alignment is not restricted to *f*_o_, but may involve multiple spectral components, potentially enhancing signal amplitude across a broader frequency range.

Our results also fit into a broader comparative framework. In birds, this form of articulatory control is well established. Northern cardinals (*Cardinalis cardinalis*), for instance, actively adjust the volume of their oropharyngeal-oesophagal cavity (OEC) during song to align its primary resonance with *f*_o_, thereby improving sound transmission efficiency [29]. Similarly, white-throated sparrows (*Zonotrichia albicollis*) coordinate movements of the larynx and hyoid apparatus to maintain a constant OEC volume during constant-frequency notes [30]. In addition to these mechanisms, some bird species also exploit tongue movements to modulate vocal tract filtering, for example, by altering resonance frequencies through changes in lingual position [95,96], highlighting the contribution of multiple articulators to acoustic control in this clade. Among mammals, grey wolves align *f*_o_ with the first vocal tract resonance during howling [22], and grasshopper mice enhance long-distance calls by tuning *f*_o_ to a higher resonance [23]. These cases suggest that dynamic vocal-tract modulation is widespread and helps shape signal intensity and transmission.

A similar pattern has been proposed in other singing primates. Haimoff [50] observed that silvery gibbons open their mouths wider when producing higher-frequency notes. Koda and colleagues [24] showed that white-handed gibbons can modify vocal-tract configuration to increase sound intensity through F1 tuning. Notably, these adjustments appear to rely primarily on changes in vocal tract configuration driven by jaw and lip movements, rather than fine-grained tongue modulation, which is thought to be relatively constrained in nonhuman primates [97,98]. Given the independent evolution of singing behaviour in primates and the considerable phylogenetic distance between lemurs and lesser apes [34], our results support the idea that vocal tract tuning may be an adaptive trait co-evolved with song. As territorial spacing represents the primary function of song emission in primates [33], the ability to increase sound intensity through vocal tract adjustments can allow them to reach a broader audience of potential receivers.

Our analyses also indicate that this capacity is not sex-specific. Although previous studies have reported some sex-related differences in indri song [39,67,68], we found no effect of sex on the relationship between mouth opening and *f*_o_. This suggests that vocal-tract modulation is a species-level feature shared by both males and females, consistent with both sexes participating in song and using it for territorial communication.

At the same time, our comparison with Gamba et al. [65] suggests that jaw opening should not be treated as the sole articulatory factor. Their study noted differences between modelled and natural formants, especially when the mouth was barely open. They proposed that lip protrusion, air-sac effects, and details of vocal-tract segmentation may also contribute to the acoustic pattern. Similarly, our approach measures only vertical mouth opening from 2D video and cannot directly capture other relevant articulatory dimensions. Together, the two studies suggest jaw opening is important for indri vocal-tract modulation, but it is probably not the only factor.

More broadly, our data provide frame-level, quantitative evidence from synchronised video and audio. They show that vertical mouth opening covaries with *f*_o_ in singing indris. By demonstrating this relationship continuously, rather than across discrete mouth-opening categories, our study strengthens earlier evidence for vocal-tract flexibility and possible formant tuning in this species.

## Conclusion

This study improves our understanding of formant tuning in singing primates. It also provides a practical way to measure articulatory behaviour directly from video recordings of wild individuals. By partially automating data extraction, the method makes it more feasible to analyse larger archival and field datasets. More generally, because the workflow can be adapted to other species and morphologies, it offers an encouraging path for broader comparative studies of vocal-tract modulation across taxa.

## Supporting information

S1 Table Distribution of frames and videos across individuals included in the final dataset. (DOCX)

S1 File Supplementary code and data. Zip archive containing the R, Python, and Praat scripts, and the associated datasets, used in the analysis (see enclosed README for details on file contents and how to reproduce the analysis).
(ZIP)
