# Supplementary material for: Dynamics of jaw kinematics and fundamental frequency suggest vocal tract tuning in the indris’ song
Source: PLoS Comput Biol. 2026 Sep 21;22(9):e1014781. doi: 10.1371/journal.pcbi.1014781 (PMC13592610; doi:10.1371/journal.pcbi.1014781)
Supplement: Supplementary file 1 [file pcbi.1014781.s001.docx]

| **Ind_ID** | **num_frames** | **num_video** | **sex** |
| --- | --- | --- | --- |
| befosty | 82 | 1 | f |
| bemasoandro | 1165 | 2 | f |
| bevolo | 153 | 1 | f |
| jery | 119 | 2 | m |
| jonah | 271 | 1 | m |
| kara | 238 | 2 | f |
| mahagaga | 115 | 2 | m |
| max | 254 | 2 | m |
| mena | 563 | 4 | f |
| tonga | 17 | 1 | f |
| tsferana | 478 | 1 | f |
| willy | 143 | 1 | m |

S1 Table – Distribution of frames and videos across individuals included in the final dataset.
