# Supplementary material for: Dynamics of jaw kinematics and fundamental frequency suggest vocal tract tuning in the indris’ song
Source: PLoS Comput Biol. 2026 Sep 21;22(9):e1014781. doi: 10.1371/journal.pcbi.1014781 (PMC13592610; doi:10.1371/journal.pcbi.1014781)
Supplement: Supplementary file 3 [file pcbi.1014781.s004.docx]

**Reviewers' comments:**
Reviewer's Responses to Questions

**Comments to the Authors:
Please note here if the review is uploaded as an attachment.**

Reviewer #1: This is an interesting analysis of vertical mouth movements and peak frequency production in Indri's. By using recordings of wild animals combined with DeepLabCut to trace the precise movements, the authors manage to convincingly show vocal tuning in a primate species. The manuscript is overall well written, the methodology is clearly explained, and the results convincingly support the main conclusions. The integration of pose estimation with acoustic analysis makes it a novel approach.

We are glad that Reviewer #1 appreciated our MS and are grateful for the helpful comments. We revised our MS in accordance with Reviewer #1's comments. We hope to have addressed all major concerns raised and that the current version of the manuscript will be acceptable for publication.

To improve the manuscript, I would recommend the following changes:

Introduction

The introduction is well written and gives clear background on vocal tuning and primate vocal production. What could be addressed more is the fact that vocal tuning is not just a mammalian trait. The authors mention this once or twice in a short sentence, but the paragraph that focuses on non-human vocal tuning is nearly completely focusing on primates. I would suggest to add an example of a non-primate mammal and a member from the bird clade (for example Riede et al., 2009, PNAS).

Thank you for this comment. We expanded the introduction section as follows (lines 119-131):

“For instance, grey wolves (*Canis lupus hudsonicus*) can modify their mouth configuration during howling by lowering the jaw, thereby aligning the fundamental frequency (*f*_o_) with the first vocal tract resonance [22]. Similarly, grasshopper mice (Onychomys spp.) are able to tune *f*_o_ to the second resonance, thereby increasing the acoustic power of their long-distance calls [23]. Interestingly, the wapiti (Cervus canadensis) exhibits modulation of the source signal by a vocal tract resonance during the production of its biphonic, whistle-like sexual calls (“bugles”), suggesting dynamic adjustment of the supralaryngeal vocal tract configuration during vocal emission [28]. Beyond mammals, formant tuning has been described in several passerine species, including the Northern cardinal (*Cardinalis cardinalis*) and the white-throated sparrow (*Zonotrichia albicollis*) [29-30]. These studies demonstrate that birds can actively regulate the volume of the oropharyngeal-oesophagal cavity (OEC) through coordinated movements of the larynx and hyoid apparatus. Such adjustments modify the primary vocal tract resonance relative to the fundamental frequency (*f*_o_), thereby enhancing acoustic transmission efficiency [29-30].”

Methods

Many datapoints of the original 19 recorded individuals, less than 10% of these recordings where actually used in the final analysis. I think it is important to mention the distribution of the retained dataset. It is later mentioned that there were 23 recordings of 12 individuals used. How are those recordings distributed across individuals? Where there certain individuals that contributed more to the dataset than others? If this final set, for examples, leans heavily on one or two well-recorded individuals, this would raise concerns for the strength of the generalisation.

We thank the reviewer for raising this point regarding the distribution of the retained dataset across individuals. The final dataset included 23 recordings from 12 individuals (7 females and 5 males). The contribution of each individual varied due to differences in recording quality and the visibility of the relevant anatomical features in the videos. The number of frames retained per individual ranged from 17 to 1165. Two individuals contributed a larger number of frames (Bemasoandro: 1165 frames from 2 videos; Mena: 563 frames from 4 videos), whereas the remaining individuals contributed between 17 and 478 frames. However, it is important to point out that, to account for the non-independence of observations within individuals and the unequal contribution of subjects, individual identity (Ind_ID) was included as a random effect in the statistical model. This approach controls for potential bias arising from repeated measurements from the same individuals and ensures that the results are not driven by a small number of well-recorded subjects. To clarify the dataset's distribution, we have now added a table (Table SM1) in the supplemental materials that reports the number of recordings and frames retained for each individual.

I think the methods would benefit from a better explanation of the rolling mean normalisation that was done for the interocular distance. For example, it is unclear why this particular threshold was chosen and how this normalisation affected the spread of vertical mouth opening values.

We thank the reviewer for this comment, and we agree that the rationale for the rolling mean window required further clarification. The 30-frame window (±15 frames around each target frame, corresponding to approximately ±0.6 s) was chosen as a compromise between two methodological considerations.

First, a sufficiently large window helps compensate for occasional missing values in the interocular distance, which may occur when landmark predictions do not exceed the likelihood cut-off during pose estimation. In some cases, individual frames may appear blurred or poorly detected even when adjacent frames are correctly identified. Using a rolling window, therefore, increases the probability of retrieving reliable neighbouring estimates of the interocular distance and reduces the loss of usable frames in the final dataset.

Second, we aimed to ensure that the smoothing procedure remained within the temporal scale of a single vocal unit. Manual inspection of the videos suggested that indris generally maintain a relatively stable head position during the emission of a single note, whereas head movements more often occur between different notes within the same phrase, possibly facilitating sound propagation in the environment. Individual notes in indri songs typically last between 1 and 4 seconds (Pollock, 1986); therefore, a ±0.6 s window remains well within the duration of a single note. This approach allowed us to stabilise the scaling reference while ensuring that the anatomical measurements remained temporally associated with the same vocal unit. Using substantially longer windows could instead incorporate frames associated with different notes or silent intervals. To ensure that the interocular distance provided a reliable scaling reference within each video, we also evaluated its coefficient of variation (CV). The CV was used as an indicator of the stability of the apparent head size across frames. When the CV did not exceed 10%, the frame-specific rolling mean was used for normalisation. When the CV exceeded this threshold, indicating greater variability likely due to changes in head orientation or camera perspective, we used the average rolling mean across the entire video as the normalisation factor to avoid introducing frame-level scaling artefacts.

We modified the methods section as follows (lines 304-317):

“As the interocular distance was not available for all frames in which we had both vertical mouth opening and fo, we computed the rolling mean of the interocular distance over a 30-frame window (15 frames before and after each target frame, i.e. 0.6 s before and after a target frame) using the rollapply() function from the package zoo [81]. This window size was chosen as a compromise between increasing the probability of retrieving reliable neighbouring estimates of the interocular distance and maintaining the smoothing procedure within the temporal scale of a single vocal unit. Individual notes in indri songs typically last between 1 and 4 s [82, 36]; therefore, a ±0.6 s window remains well within the duration of a single note. We normalized the vertical mouth opening with the rolling mean of the interocular distance, but only for cases where the coefficient of variation (hereafter, CV) for the interocular distance did not exceed 10% within the same video. When the CV exceeded 10%, indicating greater variability in apparent head size likely caused by changes in head orientation or camera perspective, we used the average rolling mean as the normalization factor. We excluded from further analyses those frames for which interocular distance was unavailable within a 30-frame window. “

It would be good to specify the auditory sampling rate here.

We thank the reviewer for pointing this out. We have now specified the audio sampling rate of the recordings in the Methods section. Audio tracks were extracted from the video files and analysed as .WAV files with their original sampling rate (48000 hz). See Lines 279

Regarding the statistics, I think it is also good to clarify why this particular distribution fitted the data best. Please explain or refer to other examples why in this case a beta distribution is more suitable than gamma or lognormal models.

Thank you for your comment. Prior to model fitting, we explored the distribution of the response variable (fundamental frequency, *f_o_*) using graphical inspection and descriptive statistics (*plotdist* and *descdist* functions from the *fitdistrplus* package), that indicated that the beta distribution was the best option for our data. We rescaled *f_o_* to fall within the (0,1) interval before model fitting for analysis. See Lines 363-365

Where random slopes included in the GLMM? If so, please clarify which and why or why you did not include the random slopes in this particular model.

Thank you for this comment. Random slopes were not included in the model. The number of observations per individual and per file was relatively limited and unevenly distributed, which can lead to overparameterization and convergence issues when fitting more complex random-effects structures. In this context, including random intercepts provided a parsimonious way to account for the hierarchical structure of the data while maintaining model stability.

Results

Clear, but quite concise. I'm wondering if anything is missing here or whether this is all the data that could be distilled from the recordings.

We thank the reviewer for this comment. Our intention in the Results section was to focus on the main findings of the statistical analysis derived from the most reliable measurements extracted from the videos. During the exploratory phase of the analysis, we also attempted to derive additional morphometric measures of mouth configuration (e.g. mouth width and polygon-based estimates of mouth area) from the tracked landmarks. However, these measurements required a larger number of consistently detected landmarks across frames. In our field recordings, the number of frames in which the DeepLabCut model predicted all relevant landmarks with sufficiently high likelihood was relatively limited. As a result, these additional metrics could only be computed for a substantially smaller subset of the dataset. To avoid introducing biases related to uneven data availability and to maximise the number of usable observations, we therefore focused our analyses on vertical mouth opening, which was the most consistently detected and reliable measure across recordings.

Discussion

In the discussion the authors connect the results well to examples from birds and mammals, I think was really well done and only think the primate section could be strengthened a bit by explaining the vocal tract morphology of the indri's and explain how this could influence the feasibility of the tuning.

Thank you for this comment. We modified the text as follows (lines 469-474):

“This finding is consistent with earlier investigations by Gamba and colleagues [65], who reported that notes produced with wider mouth openings had higher average *f_o_* and higher formant values, especially the first formant (F1, which is a resonance frequency of the vocal tract). Together, these results support the view that indris modify the supralaryngeal vocal tract (the part of the throat and mouth above the vocal cords) during song production and that increased jaw opening contributes to changes in vocal output.”

Overall it is a great study that deserves to be published. The authors did a thorough analysis and managed to retrieve interesting data from animals that are not easily recorded in the wild. Excellent and interesting.

We sincerely thank the reviewer for this positive and encouraging comment. We are particularly pleased that the reviewer recognized the effort involved in collecting recordings from animals that are challenging to study in natural conditions, as well as the value of the analyses presented in the manuscript.

######################################################################################

Reviewer #2: Review

In this paper Carugati et al test the hypothesis if the indri is tracking fo with mouth opening as a proxy for upper vocal tract modulation. They filmed wild indri’s in Madagascar during call bouts under difficult field conditions and managed to film 19 individuals. I fully respect the limitations under which these data are collected and applaud the authors for this achievement.
They tracked face motion in the 2D projection plane using DLC – building on a previous publication in Ecol Infor 2025. Novel here is that the authors extracted the simultaneous fo in the recorded sound per image frame. The projected vertical mouth opening scaled to interorbital distance positively correlated with fo, suggesting that the animals track the fo with their oral cavity formats.
Overall this is an interesting paper based on an impressive, unique dataset of a rare primate recorded in the wild, expanding on Gamba et al 2011. I find the statement that the study “ introduces a novel, partially automated framework for estimating articulatory dynamics from field recordings” is an overstatement as the paper only applies DLC and does not include any new development in computational methods. As such I am not so convinced this journal is the correct outlet for this work.
The results lead to a significant biological insight.

We thank the reviewer for the careful evaluation of the manuscript and for the positive comments on the dataset's uniqueness and the biological relevance of the results. We agree that DeepLabCut itself does not represent a new computational method. Our intention was instead to emphasise the computational workflow required to apply markerless pose estimation to opportunistic field recordings of wild arboreal primates and, most importantly, to integrate these visual measurements with acoustic parameters simultaneously extracted. In this sense, the study aims to provide a multimodal analytical framework linking articulatory dynamics and acoustic structure in primate vocal production. We believe that this integrative approach is consistent with the scope of the journal, which often publishes studies that apply existing computational or analytical tools to novel biological datasets, particularly in contexts where methodological adaptation is necessary due to the constraints of field data. In the present case, the combination of markerless pose estimation, frame-level synchronisation with acoustic measurements, and normalisation procedures designed for variable recording conditions represents an approach that aligns with current methodological developments in the study of multimodal communication in primates. Moreover, several studies published in this journal have similarly applied existing analytical methods to biological questions without introducing entirely new computational algorithms (e.g., Pickard et al., 2021, Morita et al., 2021). Our work follows a similar rationale, focusing on methodological integration rather than algorithmic development.

To address the reviewer’s concern and avoid any overstatement, we have revised the wording in the abstract (lines 73:76):

“Beyond improving our understanding of vocal production in singing primates, this study combines markerless pose estimation with frame-level acoustic analysis to quantify articulatory dynamics from field recordings, providing a multimodal approach for investigating the relationship between vocal tract configuration and acoustic output in animal communication.”

Major points

1.The paper (and the Ecol Infor 2025 one ) does not touch on the significant errors that occur when projecting a 3D pose onto a 2D plane; in methods, results and discussion this topic is not included, but needs to be. Again I fully understand the authors didn’t drag a 3D video camera system into the jungle, but since the movement of a primate mandible has basically one degree of freedom compared to the skull it is relative easy to fit a 3D stick and ball skeleton onto the 2D data especially since you have annotated both lip commissures plus the nose tip/eyes plane. Rotoscoping was not so easy when Podos did beak gape estimates in birds, but there any many tools available now. But even with just the commissures and mouth tips you can rather accurately optimize to estimate the head to camera angle in 3D. That would be a true innovative aspect that would better suit this journal and give much better proxies vocal tract opening.

We thank the reviewer for this comment. We agree that projecting a 3D pose onto a 2D image plane can introduce measurement distortions, depending on the head's orientation relative to the camera, and that this should be addressed in the manuscript. In our study, measurements were obtained from opportunistic field recordings collected under challenging conditions, where animals were often located high in the forest canopy and partially occluded by vegetation. Under these circumstances, implementing a calibrated multi-camera system suitable for 3D reconstruction is not feasible. Our goal was, therefore, to develop a method to extract approximate articulatory information from single-camera field recordings. We agree that reconstructing head orientation and mandibular motion using a simplified 3D model (e.g., stick-and-ball representations fitted to facial landmarks) could further improve the estimation of vocal tract opening. However, the primary aim of the present study was not to reconstruct the three-dimensional geometry of the vocal tract, but rather to test whether a visual proxy of mouth opening covaries with fundamental frequency in a naturalistic, field-based context. In this sense, our approach prioritises robustness and applicability to opportunistic single-camera recordings over geometric reconstruction. We therefore consider the implementation of 3D modelling approaches (e.g., those based on cranial templates) a promising methodological avenue for further studies rather than a requirement for the present study. The reviewer’s suggestion to proceed with rotoscoping is certainly an intriguing one, but it falls outside the scope of the present study. We will definitely keep this valuable suggestion in mind for future work.

Furthermore, importantly, in our previous work (Carugati et al., 2025), analyses were restricted to fully visible frames in which all 13 facial landmarks were confidently detected. Due to the pronounced snout protrusion in indris, even small head rotations typically resulted in the partial or complete occlusion of one or more landmarks, leading to their exclusion from the dataset. As a result, the retained frames were strongly biased towards near-frontal views of the head. In the present study, although we focused on a reduced set of landmarks (four points defining two distances), occlusions associated with head movements still led to the exclusion of frames in which landmarks could not be reliably detected. Consequently, the analysed dataset is similarly enriched in frames with limited head rotation. We acknowledge, however, that even under these conditions, a two-dimensional projection may introduce residual perspective distortions. We have therefore explicitly addressed this limitation in the Methods (lines 278-287) and Discussion (lines 435-450) sections of the revised manuscript.

Methods (lines 294-303):

“Given that, as mentioned above, we recorded the animals at varying distances, we normalized the vertical mouth opening using the interocular distance (RightEye_Inner-LeftEye_Inner, following [64, 75]. We note that interocular distance is a two-dimensional projection of a three-dimensional anatomical structure and may vary with head orientation relative to the camera. In particular, as the head rotates away from a frontal view, the projected interocular distance is expected to decrease non-linearly, potentially affecting this normalization procedure. Given the pronounced snout morphology of indris, even moderate deviations from frontal orientation typically reduce the visibility of key landmarks. As a result, the retained dataset is enriched with frames that approximate near-frontal head orientations, thereby limiting but not eliminating the magnitude of projection-related distortions.”

Discussion (lines 461-476)

“A further limitation arises from the need to estimate the movements of a three-dimensional structure from two-dimensional video. Both mouth opening and interorbital distance were derived from 2D facial landmark projections, so they may be affected by the head's orientation relative to the camera. As the head rotates away from a frontal view, estimated distances between landmarks are expected to decrease non-linearly. This likely affects both raw measurements and normalisation. In practice, these effects were likely reduced, though not eliminated, because we excluded frames with substantial head rotation or occlusion. The retained dataset was thus biased toward near-frontal views. Normalisation by interorbital distance reduced variation due to camera distance and scale, though it did not fully correct for projection artefacts. Overall, perspective effects probably introduced more residual noise than they altered the relative variation in mouth opening across frames.Further work could address this issue by combining markerless pose estimation with three-dimensional reconstruction, head-pose estimation, or simplified cranial models. These developments would yield a more accurate and generalizable framework for extracting articulatory information from field recordings and would strengthen multimodal analyses of primate communication.”

2.The model error needs also to be presented in the context of the measures, both interorbital distance and mouth opening.

We thank the reviewer for this comment. The DeepLabCut model yielded a test RMSE of 4.55 pixels (4.36 pixels p-cutoff). In the analyzed frames, the mean interorbital distance was 45.5 ± 17.0 pixels and the mean vertical mouth opening was 50.6 ± 23.2 pixels. Thus, the model error corresponds to approximately 10% of the interorbital distance and 9% of the average mouth opening. Furthermore, the biological variability in mouth opening greatly exceeds the model error, indicating that the observed variation is unlikely to be driven by tracking noise. This clarification has been added to the Methods section (lines 248-252) and in the Discussion (lines 421-426).

Small comments

L84 Suggest to rephrase

Thank you. We rephrase it as follows (lines 110-112):

“A small number of studies have investigated whether formant tuning occurs in non-human mammals”

L90 It is not just perceived as louder, it is actually louder.

Thank you. We corrected the sentence as follows (lines 117-119):

“When the *f*_o_ or one of its harmonics lands near F1, the vocal output becomes louder and clearer, as shown in macaques”

L97 Isn’t the transition statistics one of the important components too?

We thank the reviewer for this helpful suggestion. We agree that the sequential organization of vocal units represents an important aspect of song structure. To address this point, we have revised the sentence to clarify that vocal units are organized into phrases following a hierarchical order (lines 135-137)

“Songs are distinctive series of vocalizations comprising different types of vocal units (or notes), often organized in phrases, which are modulated in frequency and uttered following a hierarchical order”

L103 cite those few; work by Fitch and colleagues.

We agree that a small number of studies have addressed phonatory mechanisms and vocal tract configuration in singing primates. We have therefore added a reference to Koda et al. (2012), which provides an example of research examining vocal production mechanisms in gibbons, to better support this statement and a more general study by Fitch (2000).

L119-120 Not a very strong argument for your question. Suggest to rephrase.

Thanks for this comment. We rephrased as it follows (Lines 156-161):

“Although current findings support the hypothesis that singing primates can adjust their mouth configurations to enhance sound propagation during song emission, quantitative analyses linking vocal tract configuration to acoustic output remain scarce. Further investigation using quantitative approaches is therefore needed to better understand the potential role of formant tuning in these species”

L160 our current investigation

Thank you. Corrected.

L161 Do you mean social unit? This is jargon that can be easily confused with e.g. any bioacoustical unit.

We thank the reviewer for pointing out this potential ambiguity. Here, we refer to different types of vocal units (i.e., note types) within the indri song, rather than to social units. To avoid confusion, we have rephrased the sentence to explicitly refer to vocal unit types (lines 199-201)

“In indris, sex-related variation in fundamental frequency may emerge depending on the type of vocal unit (i.e., note type) produced during the song [67-68].”

Methods
L176; do you have any ideas of size variation in relation to f0, e.g. from other work?

We thank the reviewer for this suggestion. In mammals, body size is known to influence vocal parameters such as fundamental frequency and formant dispersion. However, indris show relatively limited sexual size dimorphism, and previous studies have reported that sex-related variation in *f_o_* may depend more strongly on note type than on body size alone. Moreover, indris can not survive in captivity, and morphometric measurements of known individuals are therefore scarce. As a result, studies directly linking body size to vocal parameters such as *f_o_* are currently lacking for this species. Since body size measurements were not available for the individuals included in our dataset, we did not explicitly test this relationship in the present study.

L181; Did you do any TOF of sound correction? One frame of 40 ms = 13.6 m at 340 m/s, so you are only 1-2 frames off, so it wont matter much.

We thank the reviewer for raising this point. We did not apply an explicit TOF correction between the visual and acoustic signals. However, given the recording distances (approximately 10-25 m), the expected propagation delay of sound would correspond to roughly 30-70 ms, which represents approximately 1-2 frames at our video frame rate. Because this temporal offset is small relative to the temporal resolution of our measurements, we considered its effect on the alignment between mouth opening and acoustic parameters to be negligible.

L209; This pixel error needs also to be placed in the context of the measures, both interorbital distance and mouth opening.

We thank the reviewer for this comment. We modified the Methods section as it follows (lines 264-268):

“To contextualize the scale of the DLC model error, we compared it with the size of these anatomical features in the analyzed frames. In the final dataset, the mean interorbital distance was 45.5 ± 17.0 pixels, while vertical mouth opening had a mean value of 50.6 ± 23.2 pixels. Thus, the model error corresponds to approximately 9-10% of the scale of the tracked features.”

L244; lack of 3D projection error discussion

We thank the reviewer for pointing out this important methodological aspect. We modified the Method section as follows (lines 296-203):

“We note that interocular distance is a two-dimensional projection of a three-dimensional anatomical structure and may vary with head orientation relative to the camera. In particular, as the head rotates away from a frontal view, the projected interocular distance is expected to decrease non-linearly, potentially affecting this normalization procedure. Given the pronounced snout morphology of indris, even moderate deviations from frontal orientation typically reduce the visibility of key landmarks. As a result, the retained dataset is enriched with frames that approximate near-frontal head orientations, thereby limiting but not eliminating the magnitude of projection-related distortions.”

Fig 2 , I assume the sound from frame 658 to 690 is another animal. How did Praat deal with overlapping contours like this? It would help to overlay your fo extraction on the spectrogram. The one point at [~680, 1,5] seems an outlier when the animal closed its mouth during a frame with fo estimate.

We thank the reviewer for raising this point. The point around frame ~680 does not correspond to a vocalization produced by another individual. Before extracting *f*_o_ values, we manually segmented the recordings and silenced all portions of the audio that did not correspond to the target note using Praat. Therefore, the acoustic measurements were restricted to the vocalizations of the focal individual.The point visible in the figure results from a slight misalignment between the acoustic extraction and the video frame sequence. Specifically, it likely corresponds to the final portion of the preceding note, where the *f*_o_ estimate was still detected while the mouth appeared already closed in the corresponding video frame. Because the silencing procedure in Praat was performed at the level of the audio segment rather than individual video frames, a small residual portion of the note (or echo at the end of the vocalization) may still have been associated with the subsequent frame. In addition, the misalignment visible in the original figure was partly due to an error introduced during the assembly of the different graphical components of the figure. This issue has now been corrected in the revised version of the figure.

Fig 3; How can you get this segmented sequential regression line with a GLMM? This is unclear from the methods. Without lines, the relation seems more exponential,

We thank the reviewer for pointing out that the origin of the regression line in Fig. 3 was not sufficiently clear. The line shown in the figure does not represent a segmented regression. Instead, it corresponds to the marginal effect predicted by the GLMM. Specifically, the line was generated using the *plot_model()* function from the R package sjPlot (type = "eff"), which plots the predicted relationship between the response variable and the focal predictor while holding other variables constant. The points represent the observed data, whereas the line represents the model-predicted values. Because the model was fitted using a beta distribution with a logit link, the predicted relationship is not necessarily linear on the original scale of the response variable, which may explain why the relationship can appear slightly curved when the raw points are displayed without the model fit. We also added the following phrase to the Method section (lines 389-393):

“To visualize the relationship between normalized vertical mouth opening and scaled *f*_o_, we plotted the marginal effect predicted by the GLMM using the function *plot_model()* from the R package *sjPlot [91]*. Observed data points were overlaid on the predicted relationship.”

L317; Rephrase. 1) integrating pose estimation with what? 2) extraction quantitative pose estimates is not an innovation of this paper but of DLC. Just training a model in DLC does not count as that innovative anymore.

We thank the reviewer for this comment and agree that the use of markerless pose estimation tools such as DeepLabCut does not in itself represent a methodological innovation. Our intention was instead to emphasize the application of this approach to extract the visual-mechanical component of a complex behavioural display and, most importantly, to relate it to the simultaneously produced acoustic signal. More specifically, DeepLabCut was used to quantify mouth movements during song production, allowing us to link visual measurements of vocal tract configuration with acoustic parameters. This approach enables a multimodal and quantitative investigation of a complex behaviour such as indri singing and represents an innovative way to study primate vocal production. We have therefore rephrased the initial part of discussion to better reflect this point (lines 429-430):

“In this study, we used markerless pose estimation to obtain quantitative measures of vertical mouth opening during song production.”

L328-349 Depending on model performance, the projection error is likely bigger than orbit position. Results of model performance on the test dataset are not included and here the discussion lacks discussion on the errors.

We thank the reviewer for this comment. We modified the Discussion adding the following paragraph (lines lines 437-440):

“The test error of 4.55 pixels was much smaller than both the mean interorbital distance and the mouth opening in our analysed frames (45.5 and 50.6 pixels, respectively), and also smaller than the observed biological variation in mouth opening. This suggests that tracking error is unlikely to have driven the main pattern reported here.”

334. 47-49?

We thank the reviewer for pointing this out. The references cited in this sentence correspond to two separate studies (Wiltshire et al., 2023 and Carugati et al., 2025), rather than a citation range. We realize that the original formatting [93-64] may have been misleading, and we have revised the citation to clearly indicate the two references separately [93, 64].

L372; This paper did not test transmission efficiency, but -beautifully- showed that the OEC traced the fo.
I miss some discussion on what is known in tongue position in birds/primates to strengthen your argument that mouth opening is a decent proxy. Birds have an immobile tongue, but indri’s do not.

We thank the reviewer for this important comment. We agree that vocal production involves multiple articulators, including the tongue, lips, and jaw, and that some primates show considerable orofacial mobility. However, converging anatomical and experimental evidence suggests that tongue mobility in nonhuman primates is relatively constrained in its ability to shape vocal tract acoustics. In particular, the high laryngeal position and reduced pharyngeal space limit tongue deformation, with movements largely restricted to protrusion and retraction (Lieberman et al. 1969; Takemoto 2008). Consistently, vocal tract shape in species such as macaques and great apes does not appear to be substantially modified via tongue manoeuvres during vocalization, with acoustic variation more strongly linked to laryngeal and labial adjustments (Lieberman et al. 1969). Although indri-specific data on tongue kinematics are lacking, there is no indication that they depart from these general primate constraints. Their vocal output is instead likely shaped primarily by changes in vocal tract length and aperture, making mouth opening a biologically meaningful proxy for major changes in SVT configuration.

We have clarified this point in the revised manuscript and rephrased (*in italics)*:

Lines 488-510 “In birds, this form of articulatory control is well established. Northern cardinals (*Cardinalis cardinalis*), for instance, actively adjust the volume of their oropharyngeal-oesophagal cavity (OEC) during song to align its primary resonance with the fundamental frequency (*f*_o_), thereby improving sound transmission efficiency [71]. Similarly, white-throated sparrows (*Zonotrichia albicollis*) coordinate movements of the larynx and hyoid apparatus to maintain a constant OEC volume during constant-frequency notes [72]. *In addition to these mechanisms, some bird species also exploit tongue movements to modulate vocal tract filtering, for example, by altering resonance frequencies through changes in lingual position (e.g., Suthers et al. 2016; Ohms et al. 2012), highlighting the contribution of multiple articulators to acoustic control in this clade.*

*[...]*

A similar pattern has been proposed in other singing primates. Haimoff [50] observed that silvery gibbons (*Hylobates moloch*) tend to open their mouths wider when producing higher-frequency notes. Koda and colleagues [24] showed that white-handed gibbons can modify vocal-tract configuration to increase sound intensity through F1 tuning*. Notably, these adjustments appear to rely primarily on changes in vocal tract configuration driven by jaw and lip movements, rather than fine-grained tongue modulation, which is thought to be relatively constrained in nonhuman primates (Lieberman et al. 1969; Takemoto 2008).*

Given the independent evolution of singing behaviour in primates and the considerable phylogenetic distance between lemurs and lesser apes [34], our results support the idea that vocal tract tuning may be an adaptive trait co-evolved with song. As territorial spacing represents the primary function of song emission in primates [33], the ability to increase sound intensity through vocal tract adjustments can allow them to reach a broader audience of potential receivers.”

*Added references:*

- Roderick A. Suthers, John R. Rothgerber, Kenneth Kragh Jensen; Lingual articulation in songbirds. *J Exp Biol* 15 February 2016; 219 (4): 491–500. doi: <https://doi.org/10.1242/jeb.126532>:)
- Verena R. Ohms, Gabriël J. L. Beckers, Carel ten Cate, Roderick A. Suthers; Vocal tract articulation revisited: the case of the monk parakeet. *J Exp Biol* 1 January 2012; 215 (1): 85–92. doi: <https://doi.org/10.1242/jeb.064717>)
- Philip Lieberman; Primate Vocalizations and Human Linguistic Ability. *J. Acoust. Soc. Am.* 1 December 1968; 44 (6): 1574–1584. <https://doi.org/10.1121/1.1911299>
- Takemoto H (2008) Morphological analyses and 3D modeling of the tongue musculature of the chimpanzee (Pan troglodytes). Am J Primatol 70(10):966–975. https://doi.org/10.1002/ajp. 20589

######################################################################################

Reviewer #3: This is a wonderful and very important study. I congratulate the authors for the meticulous work done, and I really enjoyed reading this well-written paper. Thank you!

We are happy that Reviewer #3 appreciated our MS and we are grateful for the helpful comments. We revised our MS according to Reviewer #3's indications.

Before eventual publication, there are however a number of issues that the authors may want to address:

Given the presented corpus of data, the authors can faithfully and reliably show that mouth opening corresponds with the fundamental frequency of indris vocalizations. What they can only speculate about, but did not show (in contrast to what is stated in line 363 of the manuscript), is that indris actually apply deliberate formant tuning by aligning the first vocal tract resonance with fundamental frequency in order to enhance the acoustic levels of the produced sounds. This study would thus greatly benefit from a (simple) computational modeling approach, quantitatively showing in the model the dependency of mouth opening (or tongue height!?!) and the first resonance center frequency. I guess that even a simple 1D waveguide model à la Kelly & Lochbaum would suffice to show the observed effects. These modeling results – which were exhaustively produced for (adult) humans but never for indris – would fill the gap in the authors’ argumentation (given the nature of the study, the authors could of course not be expected to provide in vivo vocal tract resonance measurements!) that indris in fact really apply formant tuning.

Related to the above: The authors did not perform and report any spectral analysis. It is therefore not clear whether the claimed formant tuning phenomenon involved the first harmonic (i.e., the fundamental) or any of the higher harmonics in the sound. This is a crucial limitation of the study which should please be explicitly mentioned and discussed.

Following the reviewer’s suggestion, we have performed a simulation using a Kelly & Lochbaum-inspired vocal tract model. We are extremely grateful to the reviewer for suggesting this, as it has enriched and broadened the manuscript's framing. Critical information about the model has now been inserted into the manuscript, and its results and implications are also reported. It is clear that the model supports formant tuning of F1 and F3, with implications for the output sound pressure level and the broadcast of information in the forest. This provides a mechanistic basis supporting the plausibility of formant-harmonic alignment in indri vocalizations. We have now reported the new pieces of writing at:

Methods (lines 324-350)

“Vocal tract models and estimation of formant variation with respect to the fundamental frequency

The scarcity of specimens and rigorous anatomical investigations prevented a detailed, anatomically faithful reconstruction of the indri’s vocal tract. We generated a concatenated tube computational model of the indri vocal tract by measuring and manipulating two indri skull replicas. The first replica was a high-quality polyurethane resin cast of a skull found in the Analamazaotra Special Reserve in 2003. The second was a commercially available resin cast (Skulls Unlimited, Bone Clones, Replica Indri Skull SKU BC-282). We averaged the two values to proceed with the modelling. We used variable mandible positioning to estimate vocal tract cross-sectional areas at mouth openings of 0.2 cm, 1.0 cm, 2.0 cm, 3.0 cm, 4.0 cm and 5.0 cm. This was measured as the distance from the top of the right central incisors of the maxilla and the mandible. We used a non-toxic compound composed of water, salt, wheat flour, and mineral oil to fill the cavities. This created a cast simulating the vocal tract areas of an open mouth from the back of the palate. We measured the cross-sectional areas at 0.5 cm intervals from the glottis to the tract's end [83]. We also simulated the back cavities by modelling a uniform tube. This method was consistent with Milne-Edwards's drawings in Grandidier (1875) [84]. Using measurements of indri skulls coming from different locations (N = 13, mean = 103.22 mm, sd = 2.68 mm) from the Dead Animal Collection of the Tsimbazaza Zoo in Antananarivo, we estimated a variation in greater skull length of approximately 7%. Following this, we modelled the vocal tract by simulating a 10% increase in the length and cross-sectional area of the tubes. We then collected the first three formants (F1, F2, F3) from the acoustic responses of each tube using the VTAR modelling software [85, 86]. Formants were averaged by normalised mouth opening (simulated mouth opening/inter-orbital distance). This allowed us to compare estimated formants with fundamental frequencies measured from utterances with varying mouth opening. For interpretation, we calculated multiples H2 (*f*_o_*2), H3 (*f*_o_*3), H4 (*f*_o_*4), and H5 (*f*_o_*5) to determine whether formant tuning involved only the fundamental frequency or also higher harmonics. We plotted these against estimated formant variation.”

Results (lines 405- 410)

“We grouped the formants we extracted from the acoustic responses of the vocal tract models according to the different vertical mouth openings observed during the video analysis. We plotted the *f*_o_, the calculated harmonics (H2-H5), and the formants F1-F3 in Figure 4. We found significant Spearman correlations between *f*_o_ and the estimated values of F1-F3 for the corresponding vertical mouth opening positions (N = 3599; F1: rho = 0.698, p < 0.001; F2: rho = 0.039, p = 0.021; F3: rho = 0.698, p < 0.001).”

Discussion (lines 480-497)

“A limitation of our study is that we cannot directly demonstrate active or deliberate formant tuning by indris. However, the strong correlation between fo and the modelled values of F1 and F3 provides robust support for a coupling between source and filter parameters mediated by vocal tract configuration. Importantly, the modelling results demonstrate that the observed range of mouth opening is sufficient to produce systematic shifts in formant frequencies that can align with the fundamental frequency or its harmonics. The comparison with harmonic structure (H2–H5) further indicates that such alignment is not restricted to fo, but may involve multiple spectral components, potentially enhancing signal amplitude across a broader frequency range.”

It would be advantageous to have more background about why mouth opening increases the first vocal tract resonance (and thus the first formant in the radiated acoustic spectrum). I recommend to briefly review the “perturbation theory” and sensitivity functions. The paper by Story, 2006 (DOI: 10.1121/1.2151802) may be a good start for finding the necessary references and background information. Notably, while mouth opening (but more so tongue height, please mention this in the Introduction and Discussion, because tongue height has not been measured in this study) may have a direct causal influence on the first formant, the first formant frequency can be critically affected in other places along the vocal tract as well.

We thank the reviewer for this suggestion. Following the reviewer’s recommendation, we have expanded the Introduction to briefly introduce the concepts of perturbation theory and sensitivity functions (lines 86-95). We also explicitly acknowledge that articulatory parameters other than mouth opening, such as tongue height and position, can strongly influence formant frequencies. Since tongue position was not measured in the present study, we now clarify that mouth opening should be considered a proxy for vocal tract configuration rather than the sole determinant of formant variation.

“From a biomechanical perspective, the effect of mouth opening on the first vocal tract resonance can be explained by perturbation theory and sensitivity functions of the vocal tract resonator, which describe how local changes in vocal tract geometry influence resonance frequencies [8]. In particular, increasing jaw opening enlarges the cross-sectional area near the vocal tract exit, typically raising the frequency of the first resonance and thus the first formant (F1) in the radiated acoustic spectrum. Experimental studies on speech articulation confirmed this relationship, showing that F1 increases with greater jaw opening [9-10]. Articulatory parameters such as tongue height and position also strongly influence formant structure, often even more than lip opening itself, and can affect both F1 and the second formant (F2), thereby shaping vowel identity and acoustic distinctiveness [11].”

Throughout the manuscript, the term “vocal tract tuning” should be replaced by “formant tuning”, as this is the established term for human singing. It would be very helpful to add a paragraph that explains the concept of formant tuning, quoting important papers (e.g., Miller and Schutte, 1990, DOI: 10.1016/S0892-1997(05)80018-9; Carlsson and Sundberg, 1992, DOI: 10.1016/S0892-1997(05)80150-X; Miller, 2008, ISBN: 978-0-9755307-5-7), and foremost the landmark paper by Sundberg, which seems to be especially relevant for this study (Sundberg, J. 1975. “Formant Technique in a Professional Female Singer.” Acustica 32: 89–96.).

We thank the reviewer for this helpful clarification. We agree that formant tuning is the more established term in the context of singing research. Accordingly, we have replaced the term "vocal tract tuning" with "formant tuning" throughout the manuscript. In addition, we have expanded the Introduction to briefly define the concept of formant tuning and to reference the suggested literature on the topic (lines 105-110):

“This phenomenon, known as formant tuning or vocal tract tuning, refers to the deliberate alignment of a vocal tract resonance with a harmonic of the source signal in order to increase the radiated sound level. Formant tuning is a well-documented strategy in human singing, particularly in high-frequency vocal emissions, and has been described in detail in classical studies of singing acoustics [16-20]. When such alignment occurs, substantial increases in sound level can be achieved, as quantitatively demonstrated in recent studies [21].”

A bit more background about the definition of formants is required. Are they “peaks in the radiated acoustic spectrum” or “physical properties of the vocal tract”. For speech, these two concepts can safely be equated, but as fundamental frequency approximates the center frequency of the first vocal tract resonance, the conceptual difference is crucial. For adult humans (with vocal tract lengths of about 15 to 17 cm), there is a limit of about 400 Hz above which the distinction becomes important, and where – incidentally – LPC formant analysis (like done in Praat) will cease to produce meaningful results. A recent paper by Whalen et al. (DOI: 10.1121/10.0013410) expertly discusses this notion.

We thank the reviewer for raising this point and we clarify that we consider formants as resonances in the vibrating air within the vocal tract that selectively enhance specific spectral components during phonation (Fitch et al. 2025). See lines (81-84)

The metric for the periodicity of an acoustic signal is fundamental frequency. In contrast, pitch is a psychoacoustic, i.e., perceptual quantity. Despite the misnomer in Praat (calling it “pitch”), the only applicable term in the context of this study is fundamental frequency, because no perceptual data were acquired/measured. For this reason, all mentions of “pitch” should please be removed from the manuscript (and be replaced by fundamental frequency).

We thank the reviewer for this comment. We agree that “pitch” refers to a perceptual quantity, so incorrect for our present study. Following the reviewer’s suggestion, we have replaced all occurrences of “pitch” with “fundamental frequency” throughout the manuscript, except when referring to the specific name of the Praat function used for *f*_o_ extraction.


Detailed comments:

page 6, line 65 “The pioneering work of Fant [4] …” → this was established 20 years earlier by Chiba & Kajiyama (1941), with roots going back even to Helmholtz (cf. DOI 10.53830/sing.00133 )

Thank you for this comment. We modified the text to include the suggested works (lines 84-86):

“These relationships have long been recognized in acoustic theory, from early resonance models developed by Hermann von Helmholtz [5] to later articulatory-acoustic descriptions of speech production [6-7].”

page 6, line 67 “This was confirmed by Stevens' [5] studies …” → an early landmark study is that by Lindblom & Sundberg, 1971 (DOI: 10.1121/1.1912750), which should be cited here.

Thank you for this comment. We modified the text to include the suggested works (lines 92-93):

“Experimental studies on speech articulation confirmed this relationship, showing that F1 increases with greater jaw opening [9-10].”

Page 7, line 82 “Tuning F1 or higher formants to match harmonics can enhance vocal intensity …” → please refer to a recent study by Herbst & Story, 2022 (DOI: 10.1121/10.0014421), who have quantified sound level effects of formant tuning.

We thank the reviewer for this suggestion. We have now included this study and modified the phrase as follows (lines 104-110)::

“Larger mouth openings typically raise F1 frequencies, which can help align vocal tract resonances with the harmonics of fo [15]. This phenomenon, known as formant tuning or vocal tract tuning, refers to the deliberate alignment of a vocal tract resonance with a harmonic of the source signal in order to increase the radiated sound level. Formant tuning is a well-documented strategy in human singing, particularly in high-frequency vocal emissions, and has been described in detail in classical studies of singing acoustics [16-20]. When such alignment occurs, substantial increases in sound level can be achieved, as quantitatively demonstrated in recent studies [21]”

Page 7, line 84 “Many researchers asked whether vocal tract tuning is present in non-human mammals”: If “many” researchers have asked this, then more references should be provided, not just Koda et al.

Thank you. We rephrase as it follows (lines 110-111):

“A small number of studies have investigated whether formant tuning occurs in non-human mammals [22-24]”

page 7, line 90 “the vocal output is perceived as louder and clearer”: I recommend to avoid any reference to perception, because this is not part of this study and is a convoluted concept. Tuning of a vocal tract resonance to the lowest harmonic may increase overall SOUND LEVEL by more than 30 dB, as shown e.g. in Herbst & Story, 2022 (DOI: 10.1121/10.0014421)

We agree that referring to perceptual effects is not appropriate in this context. We have therefore revised the sentence as follows (lines 117-118):

“When the *f*_o_ or one of its harmonics lands near F1, the vocal output becomes louder and clearer, as shown in macaques [15].”

page 8, line 114 “..., as soprano singers, …”: It is a mis-conception by Koda et al. that only sopranos can apply formant tuning. In fact, ALL singers are capable of it.

We thank the reviewer for this clarification. We have revised the sentence to refer more generally to trained human singers rather than specifically to soprano singers. See line 149

Page 10, lines 157–163: Perhaps move to Discussion?

We thank the reviewer for this suggestion. We understand that these points could also be discussed in the Discussion section; however, we believe it is useful to present them here to clarify the interpretative framework underlying our analyses. We will also ensure that sex-related variation and its implications are explicitly addressed in the Discussion.

Page 11, line 181 “We recorded the indris at a distance ranging between 10 and 25 metres.”: From an experimental point of view this is absolutely required. However, 10 to 25 m is a large distance which most certainly had an impact on the spatial resolution of the acquired videos, reducing the observable mouth opening to a few pixels (even though the image provided in Figure 1 looks surprisingly good – probably a “best case scenario”? … the graphs in Figure 3 look much worse). It would be good to have a short discussion about spatial resolution, pixel-to-mouth-opening relations, and potentially occurring quantization errors and issues.

We thank the reviewer for this comment. Field recordings required maintaining a distance of 10-25 m in order to avoid disturbing the animals and in line with their arboreal ecology, which inevitably resulted in variation in the spatial resolution of mouth movements across videos. To account for this, we normalized mouth opening measurements using the interocular distance as an internal reference to reduce scaling differences among recordings. We also note that markerless pose estimation allows subpixel interpolation of landmark positions, which helps mitigate quantization effects when movements correspond to small pixel displacements. Nevertheless, we acknowledge that spatial resolution may introduce some degree of measurement noise, and we have added a short discussion of this limitation in the Methods section (lines 221-225):

“We recorded the indris at a distance ranging between 10 and 25 metres. Consequently, the spatial resolution of mouth movements varied across recordings depending on the distance from the subject and the framing of the face, and this factor may introduce some degree of quantization noise, which represents an inherent limitation of video-based measurements under naturalistic field conditions.”

Page 13, line 231 “extracted fo measurements …”: Please indicate the exact setting and algorithm used in Praat (autocorrelation?)

We thank the reviewer for this suggestion. Fundamental frequency was estimated using Praat’s cross-correlation algorithm (“To Pitch (cc)”) with a time step of 0.001 s, a pitch floor of 500 Hz and a pitch ceiling of 1350 Hz. The pitch contour was then smoothed and interpolated before extracting mean *f₀* values within consecutive 40-ms windows. These details have now been added to the Methods section (lines 281-283):

“We then extracted *f*_o_ measurements using the cross-correlation pitch detection algorithm in Praat, applying a frequency range of 500-1350 Hz, at an interval of 0.04 s to equal the frame count of each video (25 fps).”

page 13 line 234 “we retained 11,467 matrices by excluding those in which either fo ... could not be computed”: Particularly the “voicing threshold” setting has a great influence on the retrieval of fundamental frequency data points from noisy signals. (so need to mention algorithm settings)

We thank the reviewer for raising this point. The reduction from the initial number of frames does not result from restrictive analytical thresholds in the pitch extraction procedure. Instead, it reflects the requirement that both acoustic and visual information were available simultaneously. Many frames corresponded either to moments when the animal was not visible (or the mouth was not clearly detectable for landmark prediction with DeepLabCut) or to silent intervals between vocal units (e.g., pauses between notes, the beginning or end of recordings, or brief pauses during duet exchanges).

page 13, lines 244 and 245 (interocular distance): This seems like the best course of action in a “tricky” dataset. The authors may however want to mention that the interocular distance is a two-dimensional projection of a phenomenon in 3D. When the animals turn their heads, the IO-distance changes, and thus the normalization procedure breaks down. Small turns won’t have large effects, but because this is in essence a sine function, larger degrees of head turning will affect the normalization. This should be quantified, mentioned as a potential limitation of the study, and possible safeguards against spurious results discussed and applied.

We thank the reviewer for highlighting this point. We agree that interocular distance (as also mouth opening) represents a two-dimensional projection of a three-dimensional anatomical feature and may vary as a function of head orientation relative to the camera. In our dataset, however, the requirement that facial landmarks be clearly visible and confidently detected resulted in the exclusion of frames with substantial head rotation or occlusion. Given the morphology of the indri face and its highly protruded snout, even moderate deviations from frontal orientation typically reduce the visibility of key landmarks. As a result, the retained frames are mostly biased towards near-frontal views, which limits the magnitude of projection-related distortions. We nonetheless acknowledge that normalization by interocular distance does not fully correct for perspective effects and that larger head rotations could introduce non-linear distortions. This limitation is now explicitly discussed in the manuscript both in the Methods section and Discussion.

Methods (lines 296-303):

“We note that interocular distance is a two-dimensional projection of a three-dimensional anatomical structure and may vary with head orientation relative to the camera. In particular, as the head rotates away from a frontal view, the projected interocular distance is expected to decrease non-linearly, potentially affecting this normalization procedure. Given the pronounced snout morphology of indris, even moderate deviations from frontal orientation typically reduce the visibility of key landmarks. As a result, the retained dataset is enriched with frames that approximate near-frontal head orientations, thereby limiting but not eliminating the magnitude of projection-related distortions.”

Discussion (lines 452-477):

“A further limitation arises from the need to estimate the movements of a three-dimensional structure from two-dimensional video. Both mouth opening and interorbital distance were derived from 2D facial landmark projections, so they may be affected by the head's orientation relative to the camera. As the head rotates away from a frontal view, estimated distances between landmarks are expected to decrease non-linearly. This likely affects both raw measurements and normalisation. In practice, these effects were likely reduced, though not eliminated, because we excluded frames with substantial head rotation or occlusion. The retained dataset was thus biased toward near-frontal views. Normalisation by interorbital distance reduced variation due to camera distance and scale, though it did not fully correct for projection artefacts. Overall, perspective effects probably introduced more residual noise than they altered the relative variation in mouth opening across frames. Further work could address this issue by combining markerless pose estimation with three-dimensional reconstruction, head-pose estimation, or simplified cranial models. These developments would yield a more accurate and generalizable framework for extracting articulatory information from field recordings and would strengthen multimodal analyses of primate communication.”

Page 21, line 398 “… my analysis does not reveal any effect”: an artifact from ChatGPT, perhaps? Please rephrase (e.g., “…, the analysis presented here does not reveal …”) … same in line 402.

We thank the reviewer for pointing this out. We revised both the sentences following the reviewer’s suggestion.

page 21, line 404 “… in primates” → “… in non-human primates”

Corrected, thank you.

**Have the authors made all data and (if applicable) computational code underlying the findings in their manuscript fully available?**
The [PLOS Data policy](https://www.google.com/url?q=https://track.editorialmanager.com/CL0/https:%252F%252Fjournals.plos.org%252Fploscompbiol%252Fs%252Fmaterials-and-software-sharing/1/010f019b27ad6914-f7c54504-0058-4acc-b171-1a0d81c110c3-000000/OcLGh0Nr_qEswmZ-xGdPKlnjqw2NYf_qDFIwWUGT9nE%3D241&source=gmail-imap&ust=1766502040000000&usg=AOvVaw1OmtlKnrPUlmGD-1agPI0g) requires authors to make all data and code underlying the findings described in their manuscript fully available without restriction, with rare exception (please refer to the Data Availability Statement in the manuscript PDF file). The data and code should be provided as part of the manuscript or its supporting information, or deposited to a public repository. For example, in addition to summary statistics, the data points behind means, medians and variance measures should be available. If there are restrictions on publicly sharing data or code —e.g. participant privacy or use of data from a third party—those must be specified.

Reviewer #1: Yes

Reviewer #2: None

Reviewer #3: **No:** The Python algorithms are missing (e.g. line 213)

We thank the Reviewer for noticing the missing Python script. We included in the resubmitted supplementary materials (script_generate_matrices.py)

PLOS authors have the option to publish the peer review history of their article ([what does this mean?](https://www.google.com/url?q=https://track.editorialmanager.com/CL0/https:%252F%252Fjournals.plos.org%252Fploscompbiol%252Fs%252Feditorial-and-peer-review-process%2523loc-peer-review-history/1/010f019b27ad6914-f7c54504-0058-4acc-b171-1a0d81c110c3-000000/aiwGiJIB0es2paj2A1KJ2GSuB0NG7zGUhuEYGLZ_ayM%3D241&source=gmail-imap&ust=1766502040000000&usg=AOvVaw22WwJzjKnh9RUV1jDxx2Wd)). If published, this will include your full peer review and any attached files.


If you choose “no”, your identity will remain anonymous but your review may still be made public.


**Do you want your identity to be public for this peer review?** For information about this choice, including consent withdrawal, please see our [Privacy Policy](https://www.google.com/url?q=https://track.editorialmanager.com/CL0/https:%252F%252Fwww.plos.org%252Fprivacy-policy/1/010f019b27ad6914-f7c54504-0058-4acc-b171-1a0d81c110c3-000000/QTCHIOLqGuY__ruJV1VSz6kVmT9UWc7NuYZAnj44yOs%3D241&source=gmail-imap&ust=1766502040000000&usg=AOvVaw3j2rWdyuB7WqYYA-QJTe4e).

Reviewer #1: **Yes:** Michelle Spierings

Reviewer #2: No

Reviewer #3: No
