# Supplementary material for: Dynamics of jaw kinematics and fundamental frequency suggest vocal tract tuning in the indris’ song
Source: PLoS Comput Biol. 2026 Sep 21;22(9):e1014781. doi: 10.1371/journal.pcbi.1014781 (PMC13592610; doi:10.1371/journal.pcbi.1014781)
Supplement: Supplementary file 4 [file pcbi.1014781.s005.docx]

Reviewer's Responses to Questions

Reviewer #2: The authors did a careful job at considering all comments. I have no further suggestions or comments.

We are grateful to the reviewer for their time and effort in evaluating our manuscript, and for confirming that the revisions satisfactorily address all previous concerns.

Reviewer #3: I sincerely thank the authors for their careful consideration of my earlier remarks. As far as I am concerned, only a few minor issues remain:

One key issue concerns perturbation theory: a given resonance/formant can be affected by variation in vocal tract geometry at different positions along the tract. This means that a change in F1 is not necessarily attributable to mouth opening alone. This remains a logical gap in the authors' reasoning that cannot be resolved with the data currently available, and it should be explicitly acknowledged in the manuscript.

We are fully aligned with the Reviewer’s suggestion. Although increases in F1 are often consistent with greater mouth opening or a shortening of the effective anterior vocal tract, we acknowledge that this interpretation is not unique. According to perturbation theory, the frequency of a given resonance can be influenced by changes in vocal-tract geometry at multiple positions along the tract, not only at the oral opening. Therefore, variation in F1 cannot be unequivocally attributed to mouth aperture alone, particularly in the absence of direct articulatory measurements. This idea is now clearly reported in the revised manuscript (lines 470-474): “It should be noted, however, that according to perturbation theory a given resonance can be affected by changes in vocal-tract geometry at multiple positions along the tract. Thus, while our results are consistent with mouth opening as a driver of F1 variation, we cannot rule out additional or alternative contributions from other supralaryngeal adjustments, such as pharyngeal configuration or lip posture.”.

On the methods: I continue to urge the authors to specify the parameters of the Praat cross-correlation algorithm used for fundamental frequency estimation, in particular the voicing threshold and octave cost. These settings substantially influence the resulting fundamental frequency data, especially for input that may be partially aperiodic.

We thank the reviewer for pressing us on this point. Fundamental frequency was estimated using Praat's cross-correlation pitch detection algorithm ("To Pitch (cc)") with the following settings: time step = 0.001 s, pitch floor = 500 Hz, pitch ceiling = 1350 Hz, silence threshold = 0.03, voicing threshold = 4.5×10⁻⁵, octave cost = 1×10⁻¹³, octave-jump cost = 3.5×10⁻¹³, voiced/unvoiced cost = 1.4×10⁻⁵, with "very accurate" pitch estimation enabled and up to 15 candidates per frame. Two considerations should be made to ensure the understanding of these changes from the default Praat parameters: i) we calculate pitch on a signal that has been cleaned using the textgrid to silence non-target singers and whose fundamental frequency is prominent against background noise; ii) early reverberance and sounds between the units of the target individuals were set to 0 to ensure optimal pitch extraction. Octave cost must tolerate a fundamental frequency change between 500 and 1350 Hz. At the same time, throughout the song, the pre-processing mentioned above allows a voicing threshold that fully covers the sound pressure level variation of the individual's singing due to head turns, across potentially different durations. The pitch contour was then smoothed (Smooth: 10 Hz bandwidth), following Gamba et al. (2016), to remove potential local inconsistencies of the pitch contour. These settings have now been added to the Methods section (lines 264-269) and the full extraction script has been made available as supplementary material / in the OSF repository

“We used a Praat script [79] to silence non-target audio sequences (e.g., silence between units and utterances by non-focal individuals) based on the TextGrid annotation. We then extracted fo measurements using the cross-correlation pitch detection algorithm in Praat ("To Pitch (cc)") with the following settings: time step = 0.001 s, pitch floor = 500 Hz, pitch ceiling = 1350 Hz, silence threshold = 0.03, voicing threshold = 4.5×10⁻⁵, octave cost = 1×10⁻¹³, octave-jump cost = 3.5×10⁻¹³, voiced/unvoiced cost = 1.4×10⁻.”

Have the authors made all data and (if applicable) computational code underlying the findings in their manuscript fully available?

The PLOS Data policy requires authors to make all data and code underlying the findings described in their manuscript fully available without restriction, with rare exception (please refer to the Data Availability Statement in the manuscript PDF file). The data and code should be provided as part of the manuscript or its supporting information, or deposited to a public repository. For example, in addition to summary statistics, the data points behind means, medians and variance measures should be available. If there are restrictions on publicly sharing data or code —e.g. participant privacy or use of data from a third party—those must be specified.

Reviewer #2: No: Authors state: All relevant data are within the manuscript and its Supporting Information files", but I cant see any SI files in the revision.

We thank the Reviewer for catching this. We have now re-uploaded all SI files together with this revised manuscript, and we have verified that they are correctly linked in the submission. We apologise for the inconvenience and thank the Reviewer for flagging this.

Reviewer #3: Yes
