# Supplementary material for: Dynamics of jaw kinematics and fundamental frequency suggest vocal tract tuning in the indris’ song
Source: PLoS Comput Biol. 2026 Sep 21;22(9):e1014781. doi: 10.1371/journal.pcbi.1014781 (PMC13592610; doi:10.1371/journal.pcbi.1014781)
Supplement: Peer review history

# Peer review: Decision Letter 0

**DOI:** 10.1371/journal.pcbi.1014781.r001

**Authors:** Feilim Mac Gabhann, Dan Stowell

16 Dec 2025

Dynamics of jaw kinematics and fundamental frequency suggest vocal tract tuning in the indris’ song

PLOS Computational Biology

Dear Dr. Carugati,

Thank you for submitting your manuscript to PLOS Computational Biology. After careful consideration, we feel that it has merit but does not fully meet PLOS Computational Biology’s publication criteria as it currently stands. Therefore, we invite you to submit a revised version of the manuscript that addresses the points raised during the review process.

Please submit your revised manuscript by Feb 15 2026 11:59PM. If you will need more time than this to complete your revisions, please reply to this message or contact the journal office at ploscompbiol@plos.org. When you're ready to submit your revision, log on to https://www.editorialmanager.com/pcompbiol/ and select the 'Submissions Needing Revision' folder to locate your manuscript file.

Please include the following items when submitting your revised manuscript:

* A letter that responds to each point raised by the editor and reviewer(s). You should upload this letter as a separate file labeled 'Response to Reviewers'. This file does not need to include responses to formatting updates and technical items listed in the 'Journal Requirements' section below.

* A marked-up copy of your manuscript that highlights changes made to the original version. You should upload this as a separate file labeled 'Revised Manuscript with Track Changes'.

* An unmarked version of your revised paper without tracked changes. You should upload this as a separate file labeled 'Manuscript'.

If you would like to make changes to your financial disclosure, competing interests statement, or data availability statement, please make these updates within the submission form at the time of resubmission. Guidelines for resubmitting your figure files are available below the reviewer comments at the end of this letter

We look forward to receiving your revised manuscript.

Kind regards,

Dan Stowell

Academic Editor

PLOS Computational Biology

Feilim Mac Gabhann

Editor-in-Chief

PLOS Computational Biology

**Additional Editor Comments (if provided):**

The reviewers agree that the research presented here is of high interest. Two reviewers raise significant issues, leading to their recommendation of "major revisions". I concur with all the reviewer comments. Please note also the requirement to disclose the accompanying software code.

**Journal Requirements:**

If the reviewer comments include a recommendation to cite specific previously published works, please review and evaluate these publications to determine whether they are relevant and should be cited. There is no requirement to cite these works unless the editor has indicated otherwise.

[Note: HTML markup is below. Please do not edit.]

**Reviewers' comments:**

Reviewer's Responses to Questions

**Comments to the Authors:**

**Please note here if the review is uploaded as an attachment.**

Reviewer #1: This is an interesting analysis of vertical mouth movements and peak frequency production in Indri's. By using recordings of wild animals combined with DeepLabCut to trace the precise movements, the authors manage to convincingly show vocal tuning in a primate species. The manuscript is overall well written, the methodology is clearly explained, and the results convincingly support the main conclusions. The integration of pose estimation with acoustic analysis makes it a novel approach.

To improve the manuscript, I would recommend the following changes:

Introduction

The introduction is well written and gives clear background on vocal tuning and primate vocal production. What could be addressed more is the fact that vocal tuning is not just a mammalian trait. The authors mention this once or twice in a short sentence, but the paragraph that focusses on non-human vocal tuning is nearly completely focusing on primates. I would suggest to add an example of a non-primate mammal and a member from the bird clade (for example Riede et al., 2009, PNAS).

Methods

Many datapoints of the original 19 recorded individuals, less than 10% of these recordings where actually used in the final analysis. I think it is important to mention the distribution of the retained dataset. It is later mentioned that there were 23 recordings of 12 individuals used. How are those recordings distributed across individuals? Where there certain individuals that contributed more to the dataset than others? If this final set, for examples, leans heavily on one or two well-recorded individuals, this would raise concerns for the strength of the generalisation.

I think the methods would benefit from a better explanation of the rolling mean normalisation that was done for the interocular distance. For example, it is unclear why this particular threshold was chosen and how this normalisation affected the spread of vertical mouth opening values.

It would be good to specify the auditory sampling rate here.

Regarding the statistics, I think it is also good to clarify why this particular distribution fitted the data best. Please explain or refer to other examples why in this case a beta distribution is more suitable than gamma or lognormal models.

Where random slopes included in the GLMM? If so, please clarify which and why or why you did not include the random slopes in this particular model.

Results

Clear, but quite concise. I'm wondering if anything is missing here or whether this is all the data that could be distilled from the recordings.

Discussion

In the discussion the authors connect the results well to examples from birds and mammals, I think was really well done and only think the primate section could be strengthened a bit by explaning the vocal tract morphology of the indri's and explain how this could influence the feasibility of the tuning.

Overall it is a great study that deserves to be published. The authors did a thorough analysis and managed to retrieve interesting data from animals that are not easily recorded in the wild. Excellent and interesting.

Reviewer #2: Review

In this paper Carugati et al test the hypothesis if the indri is tracking fo with mouth opening as a proxy for upper vocal tract modulation. They filmed wild indri’s in Madagascar during call bouts under difficult field conditions and managed to film 19 individuals. I fully respect the limitations under which these data are collected and applaud the authors for this achievement.

They tracked face motion in the 2D projection plane using DLC – building on a previous publication in Ecol Infor 2025. Novel here is that the authors extracted the simultaneous fo in the recorded sound per image frame. The projected vertical mouth opening scaled to interorbital distance positively corelated with fo, suggesting that the animals track the fo with their oral cavity formats.

Overall this is an interesting paper based on an impressive, unique dataset of a rare primate recorded in the wild, expanding on Gamba et al 2011. I find the statement that the study “ introduces a novel, partially automated framework for estimating articulatory dynamics from field recordings” is an overstaments as the paper only applies DLC and does not include any new development in computationa methods. As such I am not so convinced this journal is the correct outlet for this work.

The results lead to a significant biological insight.

Major points

1.The paper (and the Ecol Infor 2025 one ) does not touch on the significant errors that occur when projecting a 3D pose onto a 2D plane; in methods, results and discussion this topic is not included, but needs to be. Again I fully understand the authors didn’t drag a 3D video camera system into the jungle, but since the movement of a primate mandible has basically one degree of freedom compared to the skull it is relative easy to fit a 3D stick and ball skeleton onto the 2D data especially since you have annotated both lip commissures plus the nose tip/eyes plane. Rotoscoping was not so easy when Podos did beak gape estimates in birds, but there any many tools available now. But even with just the commissures and mouth tips you can rather accurately optimize to estimate the head to camera angle in 3D. That would be a true innovative aspect that would better suit this journal and give much better proxies vocal tract opening.

2.The model error needs also to be presented in the context of the measures, both interorbital distance and mouth opening.

Small comments

L84 Suggest to rephrase

L90 It is not just perceived as louder, it is actually louder.

L97 Isn’t the transition statistics one of the important components too?

L103 cite those few; work by Fitch and colleagues.

L119-120 Not a very strong argument for your question. Suggest to rephrase.

L160 our current investigation

L161 Do you mean social unit? This is jargon that can be easily confused with e.g. any bioacoustical unit.

Methods

L176; do you have any ideas of size variation in relation to f0, e.g. from other work?

L181; Did you do any TOF of sound correction? One frame of 40 ms = 13.6 m at 340 m/s, so you are only 1-2 frames off, so it wont matter much.

L209; This pixel error needs also to be placed in the context of the measures, both interorbital distance and mouth opening.

L244; lack of 3D projection error discussion

Fig 2 , I assume the sound from frame 658 to 690 is another animal. How did Praat deal with overlapping contours like this? It would help to overlay your fo extraction on the spectrogram. The one point at [~680, 1,5] seems an outlier when the animal closed its mouth during a frame with fo estimate.

Fig 3; How can you get this segmented sequential regression line with a GLMM? This is unclear from the methods. Without lines, the relation seems more exponential,

L317; Rephrase. 1) integrating pose estimation with what? 2) extraction quantitative pose estimates is not an innovation of this paper but of DLC. Just training a model in DLC does not count as that innovative anymore.

L328-349 Depending on model performance, the projection error is likely bigger than orbit position. Results of model performance on the test dataset are not included and here the discussion lacks discussion on the errors.

334. 47-49?

L372; This paper did not test transmission efficiency, but -beautifully- showed that the OEC traced the fo.

I miss some discussion on what is known in tongue position in birds/primates to strengthen your argument that mouth opening is a decent proxy. Birds have an immobile tongue, but idri’s do not.

Reviewer #3: This is a wonderful and very important study. I congratulate the authors to the meticulous work done, and I really enjoyed reading this well-written paper. Thank you!

Before eventual publication, there are however a number of issues that the authors may want to address:

Given the presented corpus of data, the authors can faithfully and reliably show that mouth opening corresponds with the fundamental frequency of indris vocalizations. What they can only speculate about, but did not show (in contrast to what is stated in line 363 of the manuscript), is that indris actually apply deliberate formant tuning by aligning the first vocal tract resonance with fundamental frequency in order to enhance the acoustic levels of the produced sounds. This study would thus greatly benefit from a (simple) computational modeling approach, quantitatively showing in the model the dependency of mouth opening (or tongue height!?!) and the first resonance center frequency. I guess that even a simple 1D waveguide model à la Kelly & Lochbaum would suffice to show the observed effects. These modeling results – which were exhaustively produced for (adult) humans but never for indris – would fill the gap in the authors’ argumentation (given the nature of the study, the authors could of course not be expected to provide in vivo vocal tract resonance measurements!) that indris in fact really apply formant tuning.

Related to the above: The authors did not perform and report any spectral analysis. It is therefore not clear whether the claimed formant tuning phenomenon involved the first harmonic (i.e., the fundamental) or any of the higher harmonics in the sound. This is a crucial limitation of the study which should please be explicitly mentioned and discussed.

It would be advantageous to have more background about why mouth opening increases the first vocal tract resonance (and thus the first formant in the radiated acoustic spectrum). I recommend to briefly review the “perturbation theory” and sensitivity functions. The paper by Story, 2006 (DOI: 10.1121/1.2151802) may be a good start for finding the necessary references and background information. Notably, while mouth opening (but more so tongue height, please mention this in the Introduction and Discussion, because tongue height has not been measured in this study) may have a direct causal influence on the first formant, the first formant frequency can be critically affected in other places along the vocal tract as well.

Throughout the manuscript, the term “vocal tract tuning” should be replaced by “formant tuning” , as this is the established term for human singing. It would be very helpful to add a paragraph that explains the concept of formant tuning, quoting important papers (e.g., Miller and Schutte, 1990, DOI: 10.1016/S0892-1997(05)80018-9; Carlsson and Sundberg, 1992, DOI: 10.1016/S0892-1997(05)80150-X; Miller, 2008, ISBN: 978-0-9755307-5-7), and foremost the landmark paper by Sundberg, which seems to be especially relevant for this study (Sundberg, J. 1975. “Formant Technique in a Professional Female Singer.” Acustica 32: 89–96.).

A bit more background about the definition of formants is required. Are they “peaks in the radiated acoustic spectrum” or “physical properties of the vocal tract”. For speech, these two concepts can safely be equated, but as fundamental frequency approximates the center frequency of the first vocal tract resonance, the conceptual difference is crucial. For adult humans (with vocal tract lengths of about 15 to 17 cm), there is a limit of about 400 Hz above which the distinction becomes important, and where – incidentally – LPC formant analysis (like done in Praat) will cease to produce meaningful results. A recent paper by Whalen et al. (DOI: 10.1121/10.0013410) expertly discusses this notion.

The metric for the periodicity of an acoustic signal is fundamental frequency. In contrast, pitch is a psychoacoustic, i.e., perceptual quantity. Despite the misnomer in Praat (calling it “pitch”), the only applicable term in the context of this study is fundamental frequency, because no perceptual data were acquired/measured. For this reason, all mentions of “pitch” should please be removed from the manuscript (and be replaced by fundamental frequency).

Detailed comments:

page 6, line 65 “The pioneering work of Fant [4] …” → this was established 20 years earlier by Chiba & Kajiyama (1941), with roots going back even to Helmholtz (cf. DOI 10.53830/sing.00133 )

page 6, line 67 “This was confirmed by Stevens' [5] studies …” → an early landmark study is that by Lindblom & Sundberg, 1971 (DOI: 10.1121/1.1912750), which should be cited here.

Page 7, line 82 “Tuning F1 or higher formants to match harmonics can enhance vocal intensity …” → please refer to a recent study by Herbst & Story, 2022 (DOI: 10.1121/10.0014421), who have quantified sound level effects of formant tuning.

Page 7, line 84 “Many researchers asked whether vocal tract tuning is present in non-human mammals”: If “many” researchers have asked this, then more references should be provided, not just Koda et al.

page 7, line 90 “the vocal output is perceived as louder and clearer”: I recommend to avoid any reference to perception, because this is not part of this study and is a convoluted concept. Tuning of a vocal tract resonance to the lowest harmonic may increase overall SOUND LEVEL by more than 30 dB, as shown e.g. in Herbst & Story, 2022 (DOI: 10.1121/10.0014421)

page 8, line 114 “..., as soprano singers, …”: It is a mis-conception by Koda et al. that only sopranos can apply formant tuning. In fact, ALL singers are capable of it.

Page 10, lines 157–163: Perhaps move to Discussion?

Page 11, line 181 “We recorded the indris at a distance ranging between 10 and 25 metres.”: From an experimental point of view this is absolutely required. However, 10 to 25 m is a large distance which most certainly had an impact on the spacial resolution of the acquired videos, reducing the observable mouth opening to a few pixels (even though the image provided in Figure 1 looks surprisingly good – probably a “best case scenario”? … the graphs in Figure 3 look much worse). It would be good to have a short discussion about spatial resolution, pixel-to-mouth-opening relations, and potentially occurring quantization errors and issues.

Page 13, line 231 “extracted fo measurements …”: Please indicate the exact setting and algorithm used in Praat (autocorrelation?)

page 13 line 234 “we retained 11,467 matrices by excluding those in which either fo ... could not be computed”: Particularly the “voicing threshold” setting has a great influence on the retrieval of fundamental frequency data poins from noisy signals. (so need to mention algorithm settings)

page 13, lines 244 and 245 (interocular distance): This seems like the best course of action in a “tricky” dataset. The authors may however want to mention that the interocular distance is a two-dimensional projection of a phenomenon in 3D. When the animals turn their heads, the IO-distance changes, and thus the normalization procedure breaks down. Small turns won’t have large effects, but because this is in essence a sine function, larger degrees of head turning will affect the normalization. This should be quantified, mentioned as a potential limitation of the study, and possible safeguards against spurious results discussed and applied.

Page 21, line 398 “… my analysis does not reveal any effect”: an artifact from ChatGPT, perhaps? Please rephrase (e.g., “…, the analysis presented here does not reveal …”) … same in line 402.

page 21, line 404 “… in primates” → “… in non-human primates”

**********

**Have the authors made all data and (if applicable) computational code underlying the findings in their manuscript fully available?**

The PLOS Data policy requires authors to make all data and code underlying the findings described in their manuscript fully available without restriction, with rare exception (please refer to the Data Availability Statement in the manuscript PDF file). The data and code should be provided as part of the manuscript or its supporting information, or deposited to a public repository. For example, in addition to summary statistics, the data points behind means, medians and variance measures should be available. If there are restrictions on publicly sharing data or code —e.g. participant privacy or use of data from a third party—those must be specified.

Reviewer #1: Yes

Reviewer #2: None

Reviewer #3: **No:**  The Python algorithms are missing (e.g. line 213)

**********

PLOS authors have the option to publish the peer review history of their article (what does this mean?). If published, this will include your full peer review and any attached files.

If you choose “no”, your identity will remain anonymous but your review may still be made public.

**Do you want your identity to be public for this peer review?**  For information about this choice, including consent withdrawal, please see our Privacy Policy.

Reviewer #1: **Yes:**  Michelle Spierings

Reviewer #2: No

Reviewer #3: No

[NOTE: If reviewer comments were submitted as an attachment file, they will be attached to this email and accessible via the submission site. Please log into your account, locate the manuscript record, and check for the action link "View Attachments". If this link does not appear, there are no attachment files.]

**Figure resubmission:**

**Reproducibility:**

?>

# Author comment: Author response to Decision Letter 1

**DOI:** 10.1371/journal.pcbi.1014781.r002

27 Apr 2026

Attachment Submitted filename: Response_to_reviewers.docx

# Peer review: Decision Letter 1

**DOI:** 10.1371/journal.pcbi.1014781.r003

**Authors:** Feilim Mac Gabhann, Dan Stowell

8 Jul 2026

PCOMPBIOL-D-25-01674R1

Dynamics of jaw kinematics and fundamental frequency suggest vocal tract tuning in the indris’ song

PLOS Computational Biology

Dear Dr. Carugati,

Thank you for submitting your manuscript to PLOS Computational Biology. After careful consideration, we feel that it has merit but does not fully meet PLOS Computational Biology's publication criteria as it currently stands. Therefore, we invite you to submit a revised version of the manuscript that addresses the points raised during the review process.

Please submit your revised manuscript by Sep 07 2026 11:59PM. If you will need more time than this to complete your revisions, please reply to this message or contact the journal office at ploscompbiol@plos.org. When you're ready to submit your revision, log on to https://www.editorialmanager.com/pcompbiol/ and select the 'Submissions Needing Revision' folder to locate your manuscript file.

Please include the following items when submitting your revised manuscript:

* A letter that responds to each point raised by the editor and reviewer(s). You should upload this letter as a separate file labeled 'Response to Reviewers'. This file does not need to include responses to formatting updates and technical items listed in the 'Journal Requirements' section below.

* A marked-up copy of your manuscript that highlights changes made to the original version. You should upload this as a separate file labeled 'Revised Manuscript with Track Changes'.

* An unmarked version of your revised paper without tracked changes. You should upload this as a separate file labeled 'Manuscript'.

If you would like to make changes to your financial disclosure, competing interests statement, or data availability statement, please make these updates within the submission form at the time of resubmission. Guidelines for resubmitting your figure files are available below the reviewer comments at the end of this letter.

As the corresponding author, your ORCID iD is verified in the submission system and will appear in the published article. PLOS supports the use of ORCID, and we encourage all coauthors to register for an ORCID iD and use it as well. Please encourage your coauthors to verify their ORCID iD within the submission system before final acceptance, as unverified ORCID iDs will not appear in the published article. Only the individual author can complete the verification step; PLOS staff cannot verify ORCID iDs on behalf of authors.

We look forward to receiving your revised manuscript.

Kind regards,

Dan Stowell

Academic Editor

PLOS Computational Biology

Feilim Mac Gabhann

Editor-in-Chief

PLOS Computational Biology

**Additional Editor Comments (if provided):**

**Reviewers' comments:**

Reviewer's Responses to Questions

**Comments to the Authors:**

**Please note here if the review is uploaded as an attachment.**

Reviewer #2: The authors did a careful job at considering all comments. I have no further suggestions or comments.

Reviewer #3: I sincerely thank the authors for their careful consideration of my earlier remarks. As far as I am concerned, only a few minor issues remain:

One key issue concerns perturbation theory: a given resonance/formant can be affected by variation in vocal tract geometry at different positions along the tract. This means that a change in F1 is not necessarily attributable to mouth opening alone. This remains a logical gap in the authors' reasoning that cannot be resolved with the data currently available, and it should be explicitly acknowledged in the manuscript.

On the methods: I continue to urge the authors to specify the parameters of the Praat cross-correlation algorithm used for fundamental frequency estimation, in particular the voicing threshold and octave cost. These settings substantially influence the resulting fundamental frequency data, especially for input that may be partially aperiodic.

**********

**Have the authors made all data and (if applicable) computational code underlying the findings in their manuscript fully available?**

The PLOS Data policy requires authors to make all data and code underlying the findings described in their manuscript fully available without restriction, with rare exception (please refer to the Data Availability Statement in the manuscript PDF file). The data and code should be provided as part of the manuscript or its supporting information, or deposited to a public repository. For example, in addition to summary statistics, the data points behind means, medians and variance measures should be available. If there are restrictions on publicly sharing data or code —e.g. participant privacy or use of data from a third party—those must be specified.

Reviewer #2: **No:**  Authors state: All relevant data are within the manuscript and its Supporting Information files", but I cant see any SI files in the revision.

Reviewer #3: Yes

**********

PLOS authors have the option to publish the peer review history of their article (what does this mean?). If published, this will include your full peer review and any attached files.

If you choose “no”, your identity will remain anonymous but your review may still be made public.

**Do you want your identity to be public for this peer review?**  For information about this choice, including consent withdrawal, please see our Privacy Policy.

Reviewer #2: No

Reviewer #3: No

[NOTE: If reviewer comments were submitted as an attachment file, they will be attached to this email and accessible via the submission site. Please log into your account, locate the manuscript record, and check for the action link "View Attachments". If this link does not appear, there are no attachment files.]

**Figure resubmission:**

**Reproducibility:**

To enhance the reproducibility of your results, we recommend that authors of applicable studies deposit laboratory protocols in protocols.io, where a protocol can be assigned its own identifier (DOI) such that it can be cited independently in the future. Additionally, PLOS ONE offers an option to publish peer-reviewed clinical study protocols. Read more information on sharing protocols at https://plos.org/protocols?utm_medium=editorial-email&utm_source=authorletters&utm_campaign=protocols

# Author comment: Author response to Decision Letter 2

**DOI:** 10.1371/journal.pcbi.1014781.r004

14 Jul 2026

Attachment Submitted filename: Response_To_Reviewer.docx

# Editor report: Decision Letter 2

**DOI:** 10.1371/journal.pcbi.1014781.r005

**Authors:** Feilim Mac Gabhann, Dan Stowell

31 Aug 2026

Dear Dr De Gregorio,

We are pleased to inform you that your manuscript 'Dynamics of jaw kinematics and fundamental frequency suggest vocal tract tuning in the indris’ song' has been provisionally accepted for publication in PLOS Computational Biology.

Before your manuscript can be formally accepted you will need to complete some formatting changes, which you will receive in a follow up email. A member of our team will be in touch with a set of requests.

Please note that your manuscript will not be scheduled for publication until you have made the required changes, so a swift response is appreciated.

IMPORTANT: The editorial review process is now complete. PLOS will only permit corrections to spelling, formatting or significant scientific errors from this point onwards. Requests for major changes, or any which affect the scientific understanding of your work, will cause delays to the publication date of your manuscript.

Should you, your institution's press office or the journal office choose to press release your paper, you will automatically be opted out of early publication. We ask that you notify us now if you or your institution is planning to press release the article. All press must be co-ordinated with PLOS.

Thank you again for supporting Open Access publishing; we are looking forward to publishing your work in PLOS Computational Biology.

Best regards,

Dan Stowell

Academic Editor

PLOS Computational Biology

Feilim Mac Gabhann

Editor-in-Chief

PLOS Computational Biology

***********************************************************

# Editor report: Acceptance letter

**DOI:** 10.1371/journal.pcbi.1014781.r006

**Authors:** Feilim Mac Gabhann, Dan Stowell

PCOMPBIOL-D-25-01674R2

Dynamics of jaw kinematics and fundamental frequency suggest vocal tract tuning in the indris’ song

Dear Dr De Gregorio,

I am pleased to inform you that your manuscript has been formally accepted for publication in PLOS Computational Biology. Your manuscript is now with our production department and you will be notified of the publication date in due course.

The corresponding author will soon be receiving a typeset proof for review, to ensure errors have not been introduced during production. Please review the PDF proof of your manuscript carefully, as this is the last chance to correct any errors. Please note that major changes, or those which affect the scientific understanding of the work, will likely cause delays to the publication date of your manuscript.

Soon after your final files are uploaded, unless you have opted out, the early version of your manuscript will be published online. The date of the early version will be your article's publication date. The final article will be published to the same URL, and all versions of the paper will be accessible to readers.

For Research, Software, and Methods articles, you will receive an invoice from PLOS for your publication fee after your manuscript has reached the completed accept phase. If you receive an email requesting payment before acceptance or for any other service, this may be a phishing scheme. Learn how to identify phishing emails and protect your accounts at https://explore.plos.org/phishing.

Thank you again for supporting PLOS Computational Biology and open-access publishing. We are looking forward to publishing your work!

With kind regards,

Anitha Samidurai

PLOS Computational Biology | Carlyle House, Carlyle Road, Cambridge CB4 3DN | United Kingdom ploscompbiol@plos.org | Phone +44 (0) 1223-442824 | ploscompbiol.org | @PLOSCompBiol
